# Diarylphosphate as a New Route for Design of Highly Luminescent Ln Complexes

**DOI:** 10.3390/molecules25173934

**Published:** 2020-08-28

**Authors:** Alexey E. Kalugin, Mikhail E. Minyaev, Lada N. Puntus, Ilya V. Taydakov, Evgenia A. Varaksina, Konstantin A. Lyssenko, Ilya E. Nifant’ev, Dmitrii M. Roitershtein

**Affiliations:** 1 A.V. Topchiev Institute of Petrochemical Synthesis RAS, 119991 Moscow, Russia; alex.kalug@gmail.com (A.E.K.); mminyaev@mail.ru (M.E.M.); ladapuntus@gmail.com (L.N.P.); janiy92@yandex.ru (E.A.V.); inif@org.chem.msu.ru (I.E.N.); 2 Moscow Institute of Physics and Technology (MIPT), 141701 Dolgoprudnyi, Moscow Region, Russia; 3 N.D. Zelinsky Institute of Organic Chemistry, RAS, 119991 Moscow, Russia; 4 V.A. Kotel’nikov Institute of Radioengineering and Electronics, RAS, 141190 Fryazino, Moscow Region, Russia; 5 P.N. Lebedev Physical Institute, RAS, 119991 Moscow, Russia; taidakov@gmail.com; 6 Chemistry Department, M.V. Lomonosov Moscow State University, 119991 Moscow, Russia; kostya@xray.ineos.ac.ru; 7 National Research University Higher School of Economics, 101000 Moscow, Russia

**Keywords:** lanthanides, luminescence, crystal structure, organophosphate ligands, N-ligands

## Abstract

Organophosphate-chloride complexes [{(2,6-*^i^*Pr_2_C_6_H_3_-O)_2_POO}_2_LnCl(CH_3_OH)_4_]·2CH_3_OH, Ln = Nd (**1**), Eu (**2**), Gd (**3**), and Tb (**4**) have been obtained and structurally characterized. Their reaction with 2,2′:6′,2″-terpyridine leads to the formation of 1:1 adducts ([{(2,6-*^i^*Pr_2_C_6_H_3_-O)_2_POO}_2_LnCl(terpy)(H_2_O)_2_(CH_3_OH)], Ln = Eu (**5**), Gd (**6**), Tb (**7**) with exception of Nd, where tris-diisopropylphenylphosphate complex [{(2,6-*^i^*Pr_2_C_6_H_3_-O)_2_POO}_3_Nd) (terpy)(H_2_O)(CH_3_OH)] (**8**) was obtained due to the ligand metathesis. A bright luminescence observed for the Eu and Tb organophosphate complexes is the first example of an application of organophosphate ligands for 4f-ions luminescence sensitization. Photophysical properties of all complexes were analyzed by optical spectroscopy and an energy transfer scheme was discussed. A combination of two types of ligands into the coordination sphere (phosphate and phenanthroline) allows designing the Eu surrounding with very high intrinsic quantum yield $Q_{Eu}^{Eu}$ (0.92) and highly luminescent Ln complexes for both visible and near-infrared (NIR) regions.

## 1. Introduction

The development of highly luminescent compounds that are potentially applicable in modern optical materials is still a great challenge due to the rapidly evolving requirements of practical life [1,2]. In coordination chemistry of lanthanide elements, the key role among organic ligands has always belonged to carboxylates [3,4]. Meanwhile, related organophosphorus ligands (phosphonates, phosphinates, and phosphoric esters), which can coordinate a Ln^3+^ cation as mono- and polydentate ligands, also have great potential for the formation of complexes with 4f elements. Lanthanide complexes with organophosphate ligands should be more resistant to hydrolysis due to the higher acidity of phosphoric acids, compared with carboxylic acids. However, these complexes, and especially their crystal structures, remain virtually unexplored. There are only a few examples of the use of aryl phosphate as a ligand in the coordination chemistry of lanthanides. Various lanthanide complexes with disubstituted organophosphate ligands are currently being studied, for example, as precatalysts for various catalytic processes, including polymerization of conjugated dienes [5,6,7,8,9].

Another attractive side of aryl phosphate complexes is the presence of a conjugated system within the ligand. Such ligands are capable of efficiently absorbing electromagnetic radiation and therefore can be considered as potential “antenna ligands” for sensibilization of the lanthanide luminescence [10,11]. There are no examples of an application of organophosphate ligands for 4f-ions luminescence sensitization. Moreover, the aryl phosphate Ln complexes are an example of a system with electronic separation between the energy-donating aromatic orbital and the energy-accepting Ln^3+^ orbital via a spacer with low vibrational frequency [12]. This separation is expected to suppress the vibrational relaxation of the Eu^3+^ ion and to induce an effective energy transfer determined by the weak electronic interaction between the energy donor (aromatic ligand) and the acceptor (Eu^3+^). Quenching of the Ln^3+^ luminescence by high-energy vibrations (typically X-H oscillators, X = C, N, O) plays an important role, especially in the case of the Eu ion, owing to the energy gap law [13]. On the other hand, aryl phosphate are bulky ligands and the introduction of several ligands into the Ln^3+^ coordination sphere can lead to substantial steric hindrances. To overcome this, we used the Cl^−^ anion as the second type of the counterion. Moreover, chloride by being a single atom turns out to be a very efficient ligand dissimilarity enhancer and coordination symmetry breaker, which can boost luminescence in a significant manner [14].

Therefore, the sterically crowded bis((2,6-diisopropyl)phenyl)phosphate ligand was taken as a first example of an Ln system containing disubstituted organophosphate for design of new Ln complexes for luminescence applications.

The present article is focused on the synthesis of mononuclear heteroleptic lanthanide organophosphate complexes of Nd, Eu, Gd, Tb, and their terpyridine and phenanthroline adducts, as well as on investigations of their molecular structure and luminescence properties. Photophysical properties of these compounds are discussed based on experimental data of singlet and triplet levels, lifetimes of excited states, integrated intensities of electronic transitions parameters, radiative (k_r_) and non-radiative rate constant (k_nr_), and overall and intrinsic quantum yields ($Q_{Ln}^{Lig},\;Q_{Eu}^{Eu}).$

## 2. Results and Discussion

### 2.1. Bis-((2,6-Diisopropyl)Phenyl)Phosphate Lanthanide Complexes, Synthesis, and Photophysical Properties

**Scheme 1:**
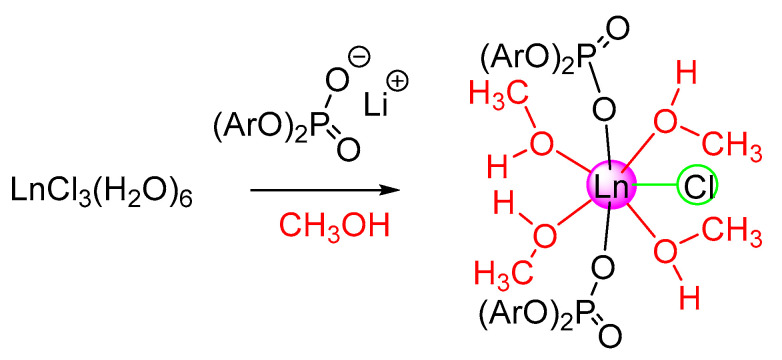
Synthesis of bisorganophosphates**1**–**4**, Ar = (2,6-diisopropyl)phenyl, Ln = Nd (**1**), Eu (**2**), Gd (**3**), and Tb (**4**).

Reactions of LnCl_3_(H_2_O)_6_ with 2 equivalents of lithium (2,6-diisopropyl) phosphate in dry methanol led to the corresponding bis(2,6-diisopropylphenyl)phosphate complexes (Scheme 1). Recrystallization of europium (**2**), gadolinium (**3**), and terbium (**4**) complexes from methanol allowed obtaining crystals suitable for single-crystal X-ray diffraction analysis.

**Figure 1:**
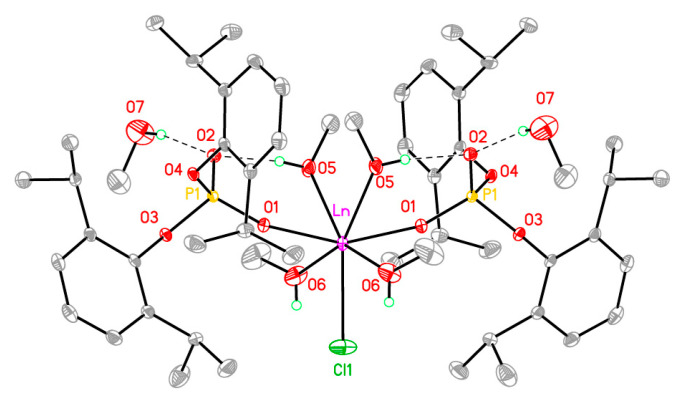
The crystal structures of **2**–**4** (using **2** as an example). Here and after, ellipsoids are drawn at 50% probability level.

The structures of **2**–**4** (Figure 1) are isomorphous with the previously described ones for complexes {[Ln(O_2_P(OAr)_2_)_2_Cl(CH_3_OH)_4_]·MeOH} (Ln = Nd (**1**), Y, Lu; Ar = O-2,6-iPr_2_C_6_H_3_) [9]. Four methanol molecules, the chloride anion, and two organophosphate ligands exhibit the κ^1^-O-terminal coordination mode (Figure 1) coordinate Ln^3+^ cation.

The closest polyhedron for the metal environment is a distorted pentagonal bipyramid with two O1 organophosphate atoms being in axial positions. The Ln-OP bond lengths are shorter by ~0.14 Å than the Ln-OMeOH ones (Table 1). Two intermolecular O_5_-H_28_···O_2_ bonds form 6-membered rings connecting the organophosphate ligand and a coordinated methanol molecule. Intermolecular OH···O hydrogen bonds (O_6_-H_29_···O_7_, O_7_-H_30_···O_2_) form a 2D OH···O hydrogen bond framework (see SI for details), which results in low solubility of these complexes in commonly used organic solvents.

### 2.2. Adducts of Bis- and Trisdiisopropylphenylphosphate Complexes with Terpyridine

**Figure 2:**
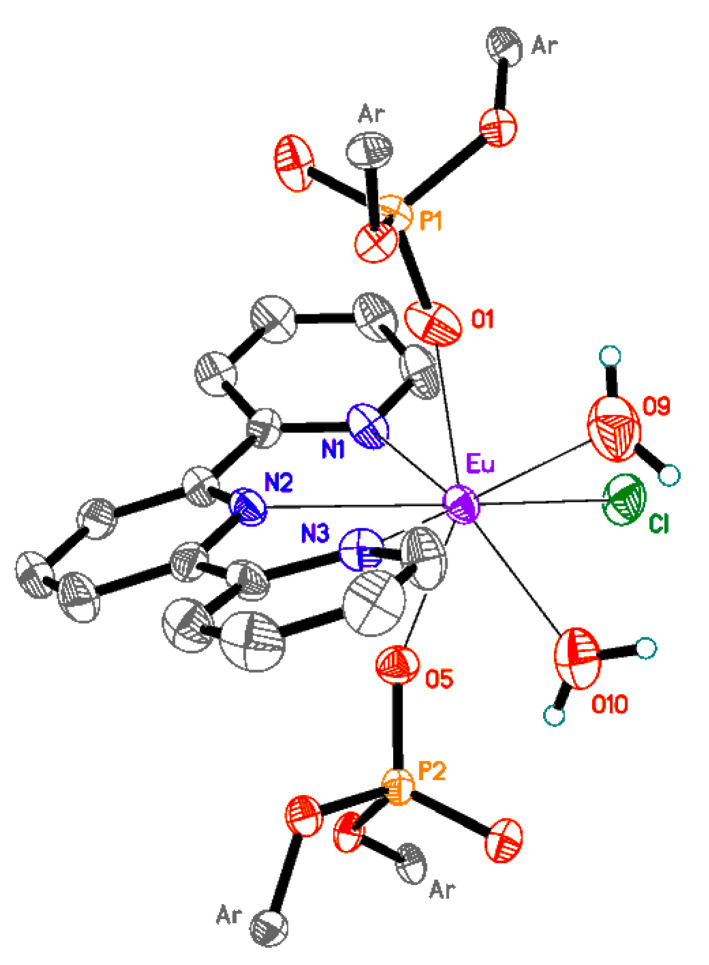
The crystal structures of **5**. A non-coordinating methanol molecule is omitted. Only ipso carbon atoms of aryl groups, labeled as Ar, are shown for clarity.

**Scheme 2:**
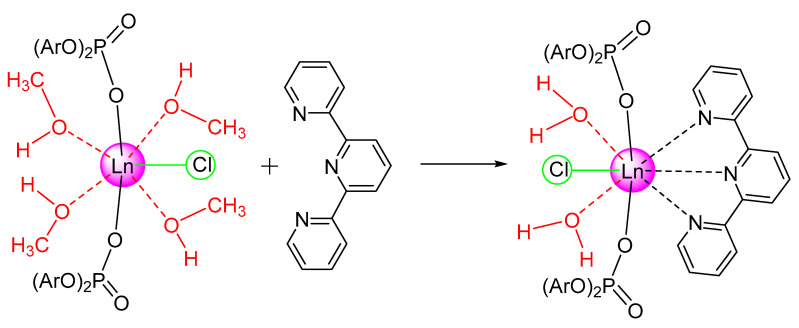
Synthesis of complexes **5**–**7**, Ln = Eu (**5**), Gd (**6**), Tb (**7**).

**Figure 3:**
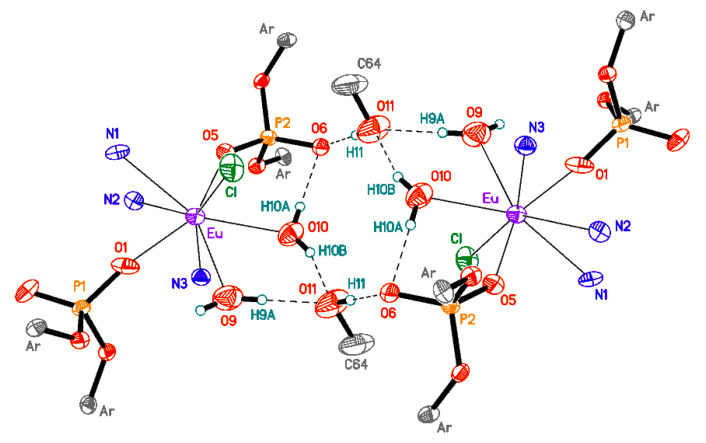
Formation of dimeric moiety {[(O_2_P(OAr)_2_)_2_EuCl(H_2_O)_2_(terpy)]_2_(MeOH)_2_} in **5** via hydrogen bonding. Carbon atoms of the terpyridine ligand are not shown.

The interaction of bis(diisopropylphenyl phosphates) **2**–**4** with an equivalent amount of terpyridine led to the formation of 1:1 adducts [{(2,6-*i*-Pr_2_C_6_H_3_O)_2_PO_2_}_2_LnCl(*ter*-pyridine)(H_2_O)_2_](CH_3_OH) Ln = Eu (**5**), Gd (**6**), and Tb (**7**) (Scheme 2). The molecular structure of **5** was resolved by X-ray studies (Figure 2 and Figure 3). We were unable to obtain X-ray quality single crystals of **6** and **7**; nevertheless, the fact that the IR spectra of complexes **5**–**7** are identical (Figures S10–S12) suggests that **6** and **7** are isostructural with **5**.

**Scheme 3:**
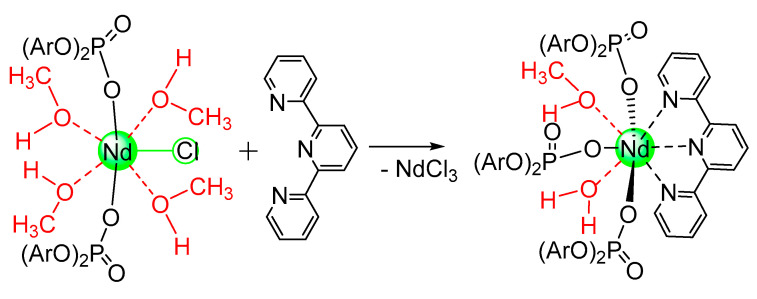
Synthesis of complex **8**.

**Figure 4:**
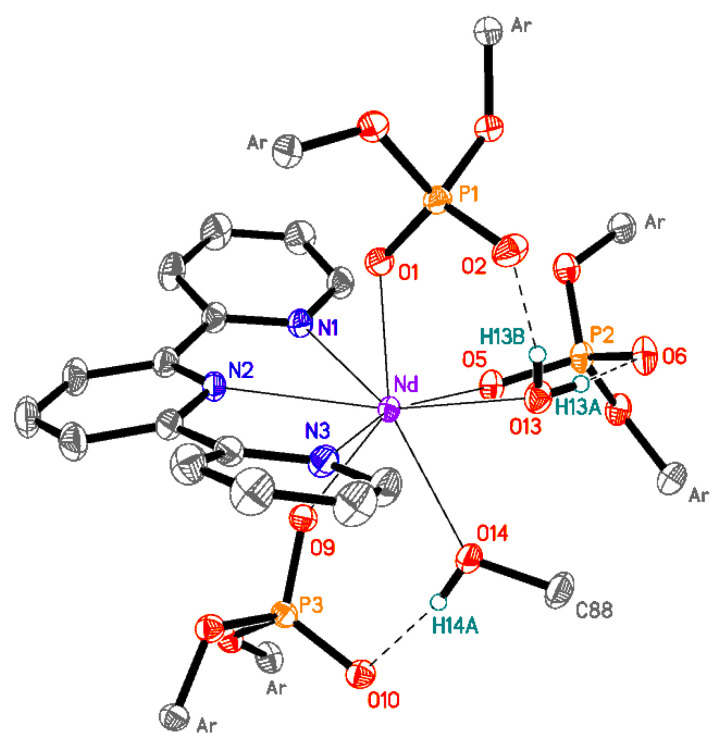
The crystal structure of **8**.

In the case of Nd complex **1**, the reaction with terpyridine was accompanied by ligands metathesis and led to the formation of a tris(diarylphosphate) complex [([{(2,6-*i*-Pr_2_C_6_H_3_O)_2_PO_2_}_3_Nd(*ter*-pyridine)(H_2_O)(CH_3_OH)] (**8**) (Scheme 3), the structure of which was also established by X-ray diffraction analysis (Figure 4). It should be noted that all terpyridine complexes contain two (complexes **5**–**7**) or one (complex **8**) water molecules coordinated with the lanthanide cation. The composition of the complexes according to X-ray diffraction data (Table S1) is in a good agreement with microanalytical data for samples taken from the bulk of the substance. The most likely source of water may be residual water in toluene. Attempts to isolate terpyridine complexes that do not contain coordinated water molecules have been unsuccessful.

Two methanol molecules present in **2** (Figure 1) are replaced in **5** (Figure 2) with a terpyridine ligand, increasing the Eu^3+^ coordination number from 7 to 8. The Eu-O_P_ bond lengths in **5** remain nearly the same (Eu-O5) or are slightly decreased by 0.04 Å (Eu-O1) as compared with Eu-O_P_ in **2** (Table 1 and Table 2), whereas the Eu-Cl bond is elongated by 0.04 Å, and the Eu-O_water_ lengths are ~0.07 Å larger than the Eu-O_MeOH_ ones in **2**. The terpyridine ligand display different dihedral angle values for the planes formed by two neighboring six-membered rings: 22.0(4)° for the ring containing N1 and 8.7(5)° for the group with N3 with respect to the middle ring. Two neighboring molecules [(O_2_P(OAr)_2_)_2_EuCl(H_2_O)_2_(terpy)] form a dimeric associate {[(O_2_P(OAr)_2_)_2_EuCl(H_2_O)_2_(terpy)]_2_(MeOH)_2_} located on an inversion center via intermolecular hydrogen bonding involving non-coordinating methanol molecules (Figure 3 and Supplementary Information).

In some respect, product **8** [(O_2_P(OAr)_2_)_3_Nd(MeOH)(H_2_O)(terpy)] (Figure 4) might be formally considered as a substitution product of complex [(O_2_P(OAr)_2_)_3_Nd(MeOH)_5_] [15], in which four coordinated methanol molecules are replaced by terpyridine and water molecules, retaining the Nd^3+^ coordination number of 8. Indeed, the interaction of [(O_2_P(OAr)_2_)_3_Nd(MeOH)_5_] with terpyridine resulted in the formation of **8** with the higher yield in comparison with the reaction of **1** with terpyridine. The Nd-O_P_ distances in **8** (Table 3) are on average shorter by ~0.02 Å than those in [(O_2_P(OAr)_2_)_3_Nd(MeOH)_5_]. The Nd-O_MeOH_ and Nd-O_water_ distances in **8** lie in the range found for Nd-O_MeOH_ bonds in [(O_2_P(OAr)_2_)_3_Nd(MeOH)_5_] (2.469(2)–2.537(2) Å, average 2.497 Å) [6]. The values of interplanar angles between neighboring rings of the terpyridine ligand are 18.53(11)° and 22.22(7)°. Complex **8** exhibits three intermolecular OH···O hydrogen bonds (Figure 4 and Supplementary Information).

Each phosphate anion in compounds **2**–**5** and **8** demonstrates that two P-O_Ar_ bonds are longer by 0.09–0.11 Å than the other two P-O distances (see SI), and the smallest O-P-O angle value corresponds to the O_Ar_-P-O_Ar_ angle between two bulky aryl ligands.

**Scheme 4:**
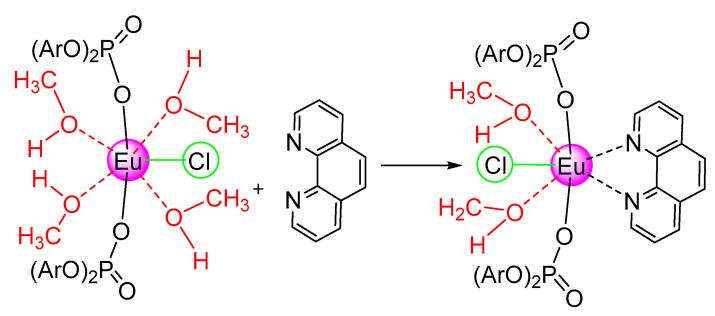
Synthesis of complex **9**.

To compare the effectiveness of various N-heterocyclic “antenna ligands”, an Eu bis (di-isopropyl phosphate)-chloride complex with phenanthroline was synthesized. The reaction of **2** with phenanthroline led to the formation of **9** (Scheme 4).

We were unable to obtain a single-crystal of **9** appropriate for X-ray analysis. Nevertheless, the spectral characteristics of the complexes prove the presence of an N-heterocyclic ligand in the coordination sphere of the Eu^3+^ cation.

### 2.3. Photophysical Properties

**Figure 5:**
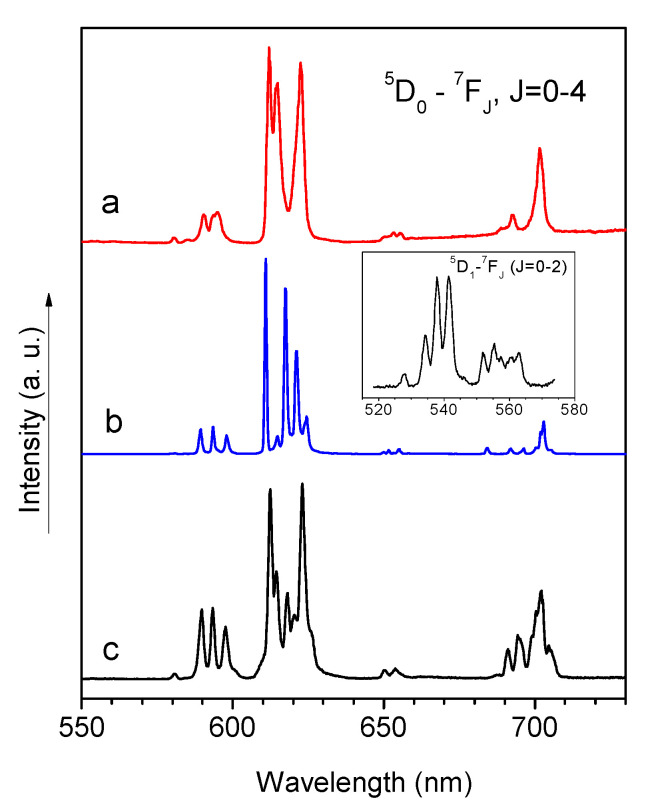
Luminescence spectra of Eu complexes **2** (a), **9** (b), and **5** (c) at 77 K, λ_exc_ = 390 for panel (a) and 340 nm for panels (b,c).

The europium ion is well known as a perfect probe for chemical surroundings, owing to its electronic configuration [16,17,18]. The luminescence spectra of the Eu complexes studied demonstrate the characteristic transitions of the Eu^3+^ ion from the excited state ^5^D_0_ to the ground state ^7^F*_J_* (*J* = 0–4) as well as from the ^5^D_1_ state in the complex **9** (Figure 5). The ^5^D_0_ → ^7^F*_J_* (*J* = 0–4) transitions display the maximum possible number of Stark components in the complexes **5** and **9**, pointing to low site symmetry for the Eu^3+^ ion, i.e., equal to or lower than *C*_2_*_ν_*. The splitting patterns of these transitions are similar in complexes **5** and **9**, while they are completely different in complex **2**. These data are confirmed by peculiarities of the coordination spheres in these complexes. Indeed, in complex **2** a coordination sphere is formed by six oxygen atoms and one chlorine atom, whereas four oxygen atoms, two nitrogen atoms, and one chlorine atom form the coordination sphere in complex **9**. Furthermore, this finding also correlates with XRD data according to which the Eu ion has different site symmetry, i.e., special position (C2) in **2** and general position in **5**. The ^5^D_0_ → ^7^F_0_ transition is strictly forbidden according to the standard Judd–Ofelt theory, and the most obvious explanation why this transition is observed is to assume that this transition is due to *J*-mixing or to mixing of low-lying charge-transfer states into the wavefunctions of the 4f^6^ configuration [16]. In the luminescence spectra, the ^5^D_0_ → ^7^F_0_ transition (region: 570–585 nm) is presented as a single symmetrical line, indicating the presence of only one type of the luminescence center in all complexes. The intensity of the magnetic dipole ^5^D_0_ → ^7^F_1_ transition is largely independent of the europium ion environment. The ratios of the integrated intensities of the europium electronic transitions to the integrated intensity of magnetic dipole transition ^5^D_0_ → ^7^F_1_ estimated from the luminescence spectra of the Eu complexes are given in Table 4. The lowest ratio obtained for the ^5^D_0_ → ^7^F_0_ transition (0.01) in complex **9** could be a result of the most symmetric charge arrangement around the Eu^3+^ ion. The highest ratio (0.05) was found in complex **2.** Assuming the data about site symmetry of the Eu^3+^ ion in **2** and **9**, the presence of the low-lying charge-transfer states can be proposed. The lowest ratio obtained for the ^5^D_0_ → ^7^F_2_ transition (3.26) in complex **5** reveals the symmetry site of the Eu^3+^ ion nearby to the inversion center. A similar value for this ratio was determined for binuclear europium pivalate and acetate with phenanthroline [19,20].

The luminescence decay curves were measured for all Eu and Tb complexes and they were fitted with a single exponential function. The lifetime values (τ) of the ^5^D_0_ emitting level are presented in Table 5. The longest τ value at 300 K between Eu complexes was found for complex **9** (1.77 ± 0.03 ms), while the shortest one was observed for the complex **2** (0.28 ± 0.01 ms).

To understand the latter, the radiative rate constant k_r_ and non-radiative rate constant k_nr_ were calculated as 4.57 × 102 s^−1^ and 31.79 × 102 s^−1^, respectively, and the large k_nr_ is probably caused by photoinduced electron transfer process (see Equation (4)). The long lifetime found in **9** can be a consequence of the absence of such effective luminescence quenchers as OH oscillators from coordinated water molecules, while the presence of coordinated methanol molecules affects it to a lesser degree, owing to the ability to form strong hydrogen bonds with PO groups and longer distance Ln-O. The shorter lifetime obtained for complex **5** (0.61 ± 0.02 ms) corresponds to the presence of four O-H oscillators coordinated as well as the increasing lifetime at 77 K (1.08 ± 0.02 ms). The longer lifetime of the Tb^3+^ ion in complex **7** in comparison with one for the Eu^3+^ ion in corresponding compound **5** (1.31 ± 0.02 vs. 0.61 ± 0.02 ms) is expected considering the energy gap law [13].

Despite the short lifetime of the Eu^3+^ ion in complex **2**, the luminescence spectrum for isostructural Tb complex was obtained. The Tb complex **4** shows the emission spectrum with typical narrow band ascribed to the terbium transitions ^5^D_4_ → ^7^F_5_ (489 nm), ^5^D_4_ → ^7^F_5_ (543 nm), ^5^D_4_ → ^7^F_4_ (585 nm), ^5^D_4_ → ^7^F_3_ (619 nm), ^5^D_4_ → ^7^F_2_ (645 nm), ^5^D_4_ → ^7^F_1_ (665 nm), and ^5^D_4_ → ^7^F_0_ (694 nm) (Figure S2). The overall quantum yield $Q_{Tb}^{Lig}$ for Tb complex **4** is equal to 10%. The Tb complex **7**, which is isostructural to Eu complex **5**, shows the emission spectrum with a typical narrow band ascribed to the terbium transitions ^5^D_4_ → ^7^F*_J_* (*J* = 5–0) (Figure S3) as well. The transition ^5^D_4_ → ^7^F_5_ is the most intense transition in this spectrum and consists 44% of total integrated intensity. The Nd complex **8** also exhibits typical luminescence with peaks centered at 900, 1080, and 1320 nm, which correspond to the ^4^F_3/2_→^4^I_9/2_, ^4^F_3/2_→^4^I_11/2_, and ^4^F_3/2_→^4^I_13/2_ transitions of the Nd^3+^ ion upon excitation through ligand absorption bands (Figure S4). The ^4^F_3/2_→^4^I_11/2_ transition is dominant and consists of ~45% of the total intensity. Therefore, the usage of diarylphosphate ligand in combination with N-heterocyclic ligands allows to obtain the strong luminescence for visible and near-infrared (NIR) regions.

As the complexes studied are potentially interesting in different optical applications such parameters as the luminance and the ways of its improvement are important. According to the concept of sensitized luminescence, energy absorbed by ligands is transferred via singlet (S_1_) and triplet (T) states to the excited levels of the lanthanide ion. In the case of the complexes containing the Tb^3+^ ion, the probability of the occurrence of the back energy transfer (BET) process is also high. Moreover, the presence of donor and acceptor fragments within a ligand can cause the charge redistribution upon the excitation and leads to intraligand charge transfer (ILCT) state. Such states were observed in the excitation spectra of lanthanide pyridine carboxylates and dicarboxylates [21,22,23]. In these complexes, the dependence of frequency of ILCT band as the function of isomer as well as on the coordination manner of carboxylate groups was discovered. The supramolecular network of H-bonds and π-stacking interactions can also induce sizeable charge redistribution within ligands and solvent molecules and affect the lanthanide surroundings. As a result of such charge redistribution, ILCT state appears and participates in the sensitization of Ln^3+^ ion luminescence. It should be noted also that in the case of Eu complexes, owing to the low redox potential of this ion, the ligand to metal charge transfer (LMCT) state may have low energy and cause the luminescence quenching [17,18]. Therefore, the energies of S_1_ and T states are very important for the optimization of intraligand energy transfer, but charge transfer states can also have a dramatic influence on the efficiency of this transfer.

**Figure 6:**
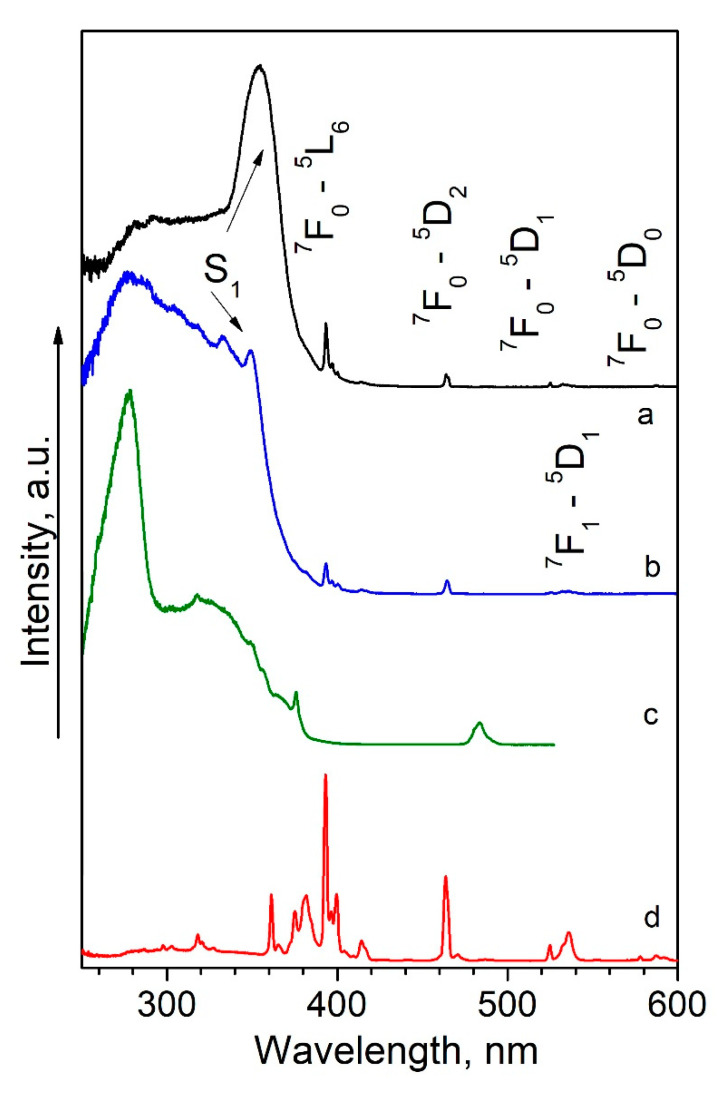
Luminescence excitation spectra of **5** (a), **9** (b), **4** (c), and **2** (d) at 300 K, λ_reg_ = 615 nm for Eu complexes (a,b,d) and 545 nm for Tb complex (c).

The luminescence excitation spectra of complexes **2** and **4** containing only organophosphate ligands exhibit narrow lines of f-f transitions of the Eu^3+^ and Tb^3+^ ions respectively (Figure 6c,d). The comparison of these excitation spectra reveals the presence of two intense bands in the region 250–380 nm in the excitation spectrum of Tb complex **4**. The first one is centered around 275 nm/36,350 cm^−1^ and can be assigned to transitions mainly attributed to the phenyl ring [24]. However, the presence of the f-d transition in this region cannot be excluded. The second broad band with a lower intensity is observed in the region 300–380 nm and its maximum was distinguished by multiple peaks fit procedure as ~325 nm/30,770 cm^−1^. As a similar band is also present in the spectrum of Eu complex **2** at 77 K (Figure S1), it can be tentatively assigned to intra- or/and interligand charge transfer due to the anion-assisted strong hydrogen bond between the coordinated methanol molecule and the P=O of the organophosphate ligand.

According to X-ray data, two intermolecular O_5_-H_28_···O_2_ bonds form 6-membered rings connecting the organophosphate ligand and coordinated methanol molecule. This hydrogen bond leads to the additional charge redistribution from anion to the neutral solvate molecule. Such charge redistribution determined by intermolecular interactions promotes the appearance of ILCT state. Similar charge redistribution caused by intermolecular H···Cl bonds, hydrogen bonds, and/or stacking interaction and resulted in additional CT state was previously observed for Ln chlorides with N-heterocyclic compounds and triflates [25]. It should be mentioned that the ligand excitation bands are almost absent in the Eu complex **2**, while both intense ligand excitation bands and the narrow bands arising from f-f transitions are present in the spectrum of terbium complex **4.** The latter indicates that complex **4** demonstrates the luminescence via direct and indirect excitation while the complex **2** only via direct excitation.

Previously, we have shown that the energy of Cl^−^ → Eu^3+^ CT state is ~340 nm in [EuCl_2_bipy_2_(H_2_O)_2_]Cl·H_2_O, where the bond length of the Eu-Cl is equal to 2.71 Å [25]. Taking into account the correlation between energy of the LMCT state and Eu-ligand bond length (the shorter the Eu-ligand bond length the shorter the wavelength of the charge-transfer band), and as the Eu-Cl bond is remarkably shorter in complex **2** (2.65 Å), one can assume the energy of LMCT state should be higher. The presence of this LMCT state can be a reason for ineffective indirect excitation observed in complex **2** and discussed above large k_nr_ also confirms this interpretation. As the energy gaps E_1_ and E_2_ are very far also from optimal (Table 5), in order to improve the efficiency of energy transfer process in Ln complexes containing two organophosphate ligands we have introduced the second type of an “antenna”, namely, terpyridine and phenanthroline ligands (complexes **5** and **9**, respectively).

**Figure 7:**
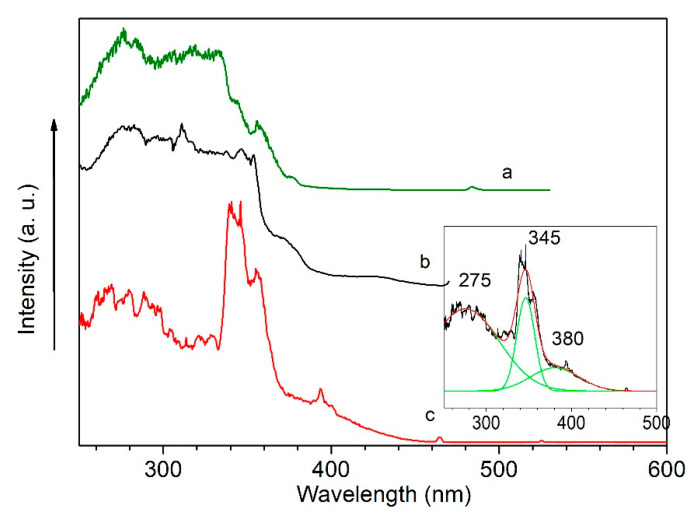
Luminescence excitation spectra of **7** (a), **6** (b) and **5** (c) at 77 K, λ_reg_ = 545 nm for (a), 490 nm for (b), and 615 nm for (c). Insert: multipeaks fit of the luminescence excitation spectrum of **5** (c) performed by Origin 8.0.

The luminescence excitation spectra of the Eu complexes **5** and **9** contain both narrow lines f-f transitions and intense broaden bands corresponding to the π-π * and n-π * transitions of heterocyclic aromatic ligands (terpyridine, phenanthroline) extending from 250 to 420 nm (Figure 6a,b). The maximum intensity of the Eu ion excitation through ligand bands is observed for the complex **9** with phen. The comparison of luminescence excitation spectra of the Eu, Gd, and Tb complexes with the terpyridine ligand (complexes **5**–**7**) reveals the presence of a broad band in the region 360–420 nm in the case of the Eu complex only (Figure 7). The maximum of this band was distinguished by multiple peaks fitting around ~380 nm/26,370 cm^−1^ nm (insert of Figure 7). The presence of this band only in the complex with the Eu ion allows its assignment to ligand → metal charge transfer state. In complex of mononuclear europium thiocyanates with terpyridine the similar excited state was not found [26] and so LMCT state observed in **5** cannot be assigned to terpyridine ligand. Taking the energy of the LMCT state as 380 nm and the electronegativity of the Eu^3+^ ion uncorrected for spin correlation as χ_uncorr_(Eu) = 1.99 [27], it was possible to estimate the optical electronegativity of the ligand (χ_opt_) participated in LMCT in **5** (see Equation (5)). The calculated value of (χ_opt_) is 2.87 for **5** that is significantly different from the value estimated for the Cl^−^→Eu^3+^ charge transfer state in [EuCl_2_bipy_2_(H_2_O)_2_]Cl·H_2_O (2.97) [25]. This difference is probably determined by cooperative effect of organophosphate ligand (having low optical electronegativity and high polarizability) and chloride anion.

The maximum of the lowest excited S_1_ state is observed at 355 and 350 nm for terpyridine and phenanthroline complexes, respectively, and it was found that the values correlate well with ones published earlier [26,27]. Structural broad band with vibronic progression ~1380 cm^−1^ attributed to stretching vibration *ν*(C=C, C=N) of phenanthroline ring observed in the region 310–350 nm of the luminescence excitation spectrum of complex **9** confirms the coordination of phenanthroline ligand. For the estimation of energy gaps E_1_ and E_2_, the energy of T state was taken as 455 nm/22,400 cm^−1^ for complex **9** [28] and as 439 nm/22,800 cm^−1^ for complex **5** from the phosphorescence spectrum of Gd complex **6** (Figure S5). Therefore, energy gap E_1_ equals to 5440 and 6370 cm^−1^ for the Eu complexes **5** and **9**, respectively (Table 5), while E_2_ is 5530 and 5000 cm^−1^ for these complexes. Importantly, in Tb complex **7**, which is isostructural to complex **5**, the E_2_ is 2330 cm^−1^ only. In this case, the estimated E_2_ value is near to limit and influence of BET process cannot be excluded. Previously BET influence on energy transfer process was demonstrated even at E_2_ ~2489 cm^−1^ [29].

The assignment of the bands in the luminescence excitation spectra along with experimentally determined energy of S_1_ and T states allows us to decipher the energy transfer process in the complexes considered. In the complexes **2**, **5**, and **9** total quantum yield ($Q_{Eu}^{Lig}$) is equal to approximately 1, 11, and 39%, respectively (Table 5). The low value of $Q_{Eu}^{Lig}$ obtained for complex **2** is reasonable taking into account that E_1_ and E_2_ gaps remarkably exceeds the optimum value (11,050 and 8100 cm^−1^, respectively) as well as the presence of ILCT and LMCT states. In complex **5**, in spite of the optimal value for effective intersystem crossing process (energy gap E_1_ is ~5440 cm^−1^), the presence of two water molecules coordinated and the low-lying LMCT state (380 nm/26,320 cm^−1^) lead to low value of intrinsic quantum yield (0.17), and therefore to low overall quantum yield $Q_{Eu}^{Lig}$. The significantly shorter lifetime of the excited ^5^D_0_ state of the Eu^3+^ ion at 300 K in comparison with one for 77 K is in line with this interpretation. The high value of $Q_{Tb}^{Lig}$ (0.45) in isostructural terbium complex **7** correlates well with the above explanation as the Tb ion is less sensitive to the presence of the O-H oscillators in the coordination sphere and LMCT state does not participate in the energy transfer process. The introduction of phosphate ligands into the coordination sphere along with phenanthroline ligand allows to design the Eu surrounding with a very high value of intrinsic quantum yield $Q_{Eu}^{Eu}$, which is 0.92 in the complex **9**. Such high value indicates that nonradiative processes are neglectable, the radiative rate constant k_r_ and non-radiative rate constant k_nr_ were calculated as 5.21 × 102 s^−1^ and 0.44 × 102 s^−1^, respectively. The high efficiency of the Eu ion excitation through ligand absorption bands observed in complex **9** along with high value of intrinsic quantum yield $Q_{Eu}^{Eu}$ lead to relatively high overall quantum yield $Q_{Eu}^{Lig}$ (0.39).

Interestingly that Eu complex with one phenanthroline ligand and chloride anions [EuCl_2_Phen(H_2_O)_4_]Cl·H_2_O studied [28] demonstrates lifetime of the ^5^D_0_ level as 0.27 ± 0.02 ms vs. 1.77 ± 0.03 ms for complex **9** ([(O_2_P(OAr)_2_)_2_EuCl(CH_3_OH)_2_(phen)]) and intrinsic quantum yield $Q_{Eu}^{Eu}$ 0.08 vs. 0.92, respectively. This comparison allows to conclude that high intrinsic quantum yield in **9** is not solely the consequence of phenanthroline ligand introduction but the combination of phosphate ligand, chloride anion, and phenanthroline that is shown to be almost ideal from electronic and steric points of view for effective sensitization of the Eu^3+^ ion luminescence.

## 3. Materials and Methods

### 3.1. General Remarks

All described manipulations were conducted under argon using standard Schlenck and vacuum line techniques. Hydrated lanthanide chlorides LnCl_3_(H_2_O)_6_ qualification “chemically pure” were used in the synthesis of complexes **1**–**4**. Argon was purified using columns with vermiculite impregnated with Mn^II^ oxide [30]. Tetrahydrofuran was predried over NaOH and distilled from potassium/benzophenone ketyl. Hexane was distilled from Na/K alloy/benzophenone ketyl. Toluene was distilled over sodium. [{(2,6-*^i^*Pr_2_C_6_H_3_-O)_2_POO}Li(MeOH)_3_]·MeOH was prepared according to the previously described procedure [15] [{(2,6-*^i^*Pr_2_C_6_H_3_-O)_2_POO}_2_NdCl(MeOH)_4_]·2MeOH (**1**) was prepared according to literature [31]. Elemental analyses were performed with a Perkin-Elmer 2400 Series II elemental CHNS/O analyzer Perkin-Elmer, Billerica, MA, USA). The analysis of the complexes **4** and **9** for the content of carbon and hydrogen provides lower carbon values, likely resulting from the incomplete combustion and/or carbides formation, repeated experiments, using independently synthesized complexes results in the same values. The high-resolution mass spectra of **9** were recorded on a SolariX XR FT/ICR mass spectrometer (Bruker Daltonic GmbH, Bremen, Germany) equipped with a 15 T superconducting magnet, a ParaCell analyzer cell, and an ESI Apollo II and MALDI SmartBeam-II ion source. External calibration of the mass spectrometer was performed using a low-concentration sodium trifluoroacetate solution (0.1 mg/mL in acetonitrile/water, 1:1). The data set size was 4M with 100 scans. All MALDI-FT-ICR-MS measurements were recorded in positive ion mode with MTP 384 ground steel target plate (3-indoleacrylic acid (IAA) matrix, with a scan range of *m/z* 150 to 3000). Twenty microliters of sample (10 μg/mL in methanol) was mixed with 20 μL of 3-indoleacrylic acid (saturated solution in THF) using a pipette and placed onto a MTP 384 ground steel target plate (Bruker Daltonics, Bremen, Germany) and allow to dry at room temperature. All data visualization was processed using Data Analysis 5.1 software package from Bruker Daltonics (Billerica MS, USA). FTIR ATR spectra were measured on a Vertex 70v IR Fourier spectrometer (Bruker Optics, Ettlingen, Germany) using a Pike ATR accessory with a diamond crystal.

### 3.2. X-ray Crystallography

Single crystals of complexes **2**–**4** were isolated from the reaction mixtures. Crystals of **5** and **8** were obtained by slow diffusion of hexane into a toluene solution of the complexes. X-ray diffraction data for all studied complexes were collected on a SMART APEX II area-detector diffractometer (graphite monochromator, ω-scan technique) using MoK_α_-radiation (0.71073 Å). The intensity data were integrated by the SAINT program [32] and were corrected for absorption and decay using SADABS [33]. All structures were solved by direct methods using SHELXS [34] and were refined on F^2^ using SHELXL [35]. All non-hydrogen atoms were refined with anisotropic displacement parameters. Positions of hydroxy and water H atoms in **2**–**4** and **8** were found from the electron density difference maps; these H atoms were refined with individual isotropic displacement parameters. All other hydrogen atoms were placed in ideal calculated positions and refined as riding atoms with relative isotropic displacement parameters taken as *U*_iso_(H) = 1.5*U*_eq_(C) for methyl groups and *U*_iso_(H) = 1.2*U*_eq_(C) otherwise. A rotating group model was applied for methyl groups. A highly disordered hexane molecule in 8 was removed by the SQUEEZE method [36]. The SHELXTL program suite was used for molecular graphics [33]. CCDC-1966199 (**2**), 1966200 (**3**), 1966201 (**4**), 1966202 (**5**), and 1966203 (**8**) contain the supplementary crystallographic data for this paper. These data can be obtained free of charge via http://www.ccdc.cam.ac.uk/conts/retrieving.html (or from the CCDC, 12 Union Road, Cambridge CB2 1EZ, UK; Fax: +44-1223-336033; E-mail: deposit@ccdc.cam.ac.uk. The crystallographic data and structure refinement details for **2**–**5** and **8** are given in Table S1 (See Supplementary Material ESI).

### 3.3. Synthesis and Characterization

#### 3.3.1. Synthesis of [{(2,6-iPr_2_C_6_H_3_-O)_2_POO}_2_EuCl(CH_3_OH)_4_]·2CH_3_OH (**2**)

[{(2,6-*^i^*Pr_2_C_6_H_3_-O)_2_POO}Li(CH_3_OH)_3_]·CH_3_OH (1.564 g (2.84 mmol)) was dissolved in 20 mL of dry CH_3_OH and dropwise added to a 10 mL of a stirred solution of 0.538 g (1.42 mmol) of EuCl_3_·6H_2_O in CH_3_OH, which led to the formation of a crystalline precipitate. The mixture was left in the freezer overnight. Several crystals were taken for X-ray studies. The precipitate was filtered off under argon, vacuum dried and recrystallized from methanol and then vacuum dried until the constant weight. The yield: 1.605 g (1.32 mmol, 93%) of 2. Anal. calculated for C_54_H_92_P_2_ClO_14_Eu: C, 53.40%; H, 7.63%. Found: C, 52.15%; H, 8.25%. Complexes **3** and **4** were prepared in a similar way to the synthesis of **2**.

#### 3.3.2. Synthesis of [{(2,6-iPr_2_C_6_H_3_-O)_2_POO}_2_GdCl(CH_3_OH)_4_]·2CH_3_OH (**3**)

Following the same procedure, 1.660 g (3 mmol) of [{(2,6-*^i^*Pr_2_C_6_H_3_-O)_2_POO}Li(CH_3_OH)_3_]·CH_3_OH, 0.588 g (1.5 mmol) of GdCl_3_·6H_2_O yielded 1.344 g (1.10 mmol, 74%). Anal. calculated for C_54_H_92_P_2_ClO_14_Gd: C, 53.16%; H, 7.60%. Found: C, 52.97%; H, 7.80%.

#### 3.3.3. Synthesis of [{(2,6-iPr_2_C_6_H_3_-O)_2_POO}_2_TbCl(CH_3_OH)_4_]·2CH_3_OH (**4**)

Following the same procedure, 1.660 g (3 mmol) of [{(2,6-*^i^*Pr_2_C_6_H_3_-O)_2_POO}Li(CH_3_OH)_3_]·MeOH and 0.560 g (1.5 mmol) of TbCl_3_·6H_2_O yielded 1.638 g (1.34 mmol, 89%). Anal. calculated for C_54_H_92_P_2_ClO_14_Tb: C, 53.09%; H, 7.59%. Found: C, 51.84%; H, 8.33%.

#### 3.3.4. Synthesis of [{(2,6-iPr_2_C_6_H_3_-O)_2_POO}_2_EuCl(terpy)(H_2_O)_2_(CH_3_OH)] (**5**)

A toluene solution of 0.070 g (0.3 mmol) of terpyridine (terpy), was slowly added to a stirred solution of 0.304 g (0.25 mmol) of **2** in toluene. The reaction mixture was heated to a boil, and then it was slowly cooled to room temperature and left in a freezer overnight. The resulting precipitate was dried in vacuum to constant weight to yield 0.261 g (0.164 mmol, 66%) of **5**. Anal. calculated for C_64_H_87_N_3_P_2_ClO_11_Eu: C, 58.07%; H, 6.62%; N, 3.17%. Found: C, 57.63%; H, 7.35%; N, 3.10%. IR (cm^−1^) 3648 (w), 3064 (w), 2958 (s), 2865 (s), 1599 (m), 1575 (m), 1453 (s), 1437 (s), 1380 (w), 1329 (w), 1257 (m), 1208 (m), 1168 (m), 1072 (s), 1012 (w), 918 (m), 904 (m), 881 (w), 770 (m), 664 (w), 475 (w). MALDI-(+)MS [M]^+^ = 1220.46074 Da, calculated for C_63_H_79_N_3_P_2_O_8_Eu = 1220.45492 Da, Δ = 4.8 ppm. C_63_H_79_N_3_P_2_O_8_Eu = (**5** − 2H_2_O-CH_3_OH − Cl^−^) (Figure S15). X-ray quality crystals were obtained by slow diffusion of hexanes into the toluene solution of **5**. Compounds **6**–**7** were synthesized in a similar way to the synthesis of **5**.

#### 3.3.5. Synthesis of [{(2,6-iPr2C6H3-O)2POO}2GdCl(terpy)(H_2_O)_2_(CH_3_OH)] (**6**)

Terpyridine (0.070 g (0.3 mmol)), 0.558 g (0.25 mmol) of 3 yielded: 0.257 g (0.193 mmol, 77%) of 6. Anal. calculated for C_64_H_87_N_3_P_2_ClO_11_Gd: C, 57.84%; H, 6.60%; N, 3.16%. Found: C, 58.87%; H, 6.56%; N, 2.96%. IR (cm^−1^) 3601 (w), 3065 (w), 2957 (s), 2866 (s), 1600 (m), 1575 (m), 1453 (s), 1437 (s), 1381 w(), 1330 (w), 1257 (m), 1210 (m), 1167 (m), 1075 (s), 1012 (w), 916 (m), 905 (m), 881 (w), 770 (m), 664 (w), 477 (w). MALDI-(+)MS [M]^+^ = 1224.45889 Da, calculated for C_63_H_79_N_3_P_2_O_8_Gd = 1224.45839 Da, Δ = 0.4 ppm. C_63_H_79_N_3_P_2_O_8_Gd = (**6** − 2H_2_O-CH_3_OH − Cl^−^). (Figure S16).

#### 3.3.6. Synthesis of [{(2,6-iPr_2_C_6_H_3_-O)_2_POO}_2_TbCl(terpy)(H_2_O)_2_(CH_3_OH)] (**7**)

Following the same procedure, 0.070 g (0.3 mmol) of terpyridine, 0.305 g (0.25 mmol) of **4** yielded 298 mg (0.187 mmol, 75%) of **7**. Anal. calculated for C_64_H_87_N_3_P_2_ClO_11_Tb: C, 57.77%; H, 6.59%; N, 3.16%. Found: C, 57.65%; H, 7.84%; N, 3.10%. IR (cm^−1^) 3641 (w), 3064 (w), 2956 (s), 2865 (s), 1599 (m), 1575 (m), 1453 (s), 1380 (w), 1329 (w), 1257 (m), 1209 (m), 1167 (m), 1076 (s), 1012 (w), 919 (m), 902 (m), 881 (w), 770 (m), 528 (w), 477 (w). MALDI-(+)MS [M]^+^ = 1226.46134 Da, calculated for C_63_H_79_N_3_P_2_O_8_Tb = 1226.45905 Da, Δ = 1.8 ppm. C_63_H_79_N_3_P_2_O_8_Tb = (**7** − 2H_2_O-CH_3_OH − Cl^−^) (Figure S17).

#### 3.3.7. Synthesis of [[{(2,6-iPr_2_C_6_H_3_-O)_2_POO}_3_Nd)(terpy)(H_2_O)(CH_3_OH)] (8)

Route A. A toluene solution of 0.070 g (0.3 mmol) of 2,2′;6′,2″-terpyridine (terpy) was slowly added to a stirred solution of 0.25 mmol of **1** in toluene. The mixture was brought to a boil, slowly cooled to room temperature, and left in a freezer overnight. The mother liquor was removed and precipitate was dried in vacuum to constant weight to yield 0.243 g (0.144 mmol, 58%) of **8**. Anal. calculated for C_88_H_119_N_3_P_3_O_14_Nd: C, 61.79%; H, 7.28%; N, 2.43%. Found: C, 61.73%; H, 7.13%; N, 2.65%. IR (cm^−1^) 2963 (s), 2927 (w), 2866 (s), 1599 (m), 1575 (m), 1327 (w), 1254 (m), 1189 (m), 1168 (m), 1069 (s), 1045 (w), 1013 (w), 931 (m), 906 (m), 771 (m), 473 (w).

Route B. A toluene solution of 0.070 g (0.3 mmol) of 2,2′;6′,2″-terpyridine (terpy) was slowly added to a stirred solution of 0.397 g (0.255 mmol) of [(O_2_P(OAr)_2_)_3_Nd(MeOH)_5_] in toluene. The mixture was brought to a boil, cooled to room temperature, and left in a freezer overnight. The precipitate formed was dried in vacuum to constant weight to yield 0.404 g (0.240 mmol, 94%) of **8**, which was identified by IR spectrum (Figure S13a), which was identical to the described above.

#### 3.3.8. Synthesis of [{(2,6-iPr_2_C_6_H_3_-O)_2_POO}_2_EuCl(phen)(CH_3_OH)_2_] (9)

Following the same procedure, 0.054 g (0.3 mmol) of o-phenantroline, 0.304 g (0.25 mmol) of **2** yielded: 198 mg (0.156 mmol, 62%) of **9**. Anal. calculated for C_62_H_84_N_2_P_2_ClO_10_Eu: C, 58.79%; H, 6.68%; N, 2.21%. Found: C, 54.51%; H, 5.63%; N, 3.10%. IR (cm^−1^) 3648 (w), 3063 (w), 2956 (s), 2865 (s), 2650 (s), 1598 (m), 1575 (m), 1453 (m), 1437 (m), 1380 (w), 1329 (w), 1257 (m), 1208 (m), 1167 (m), 1072 (s), 1012 (w), 918 (m), 903 (m), 881 (s), 769 9s), 664 (w), 528 (w), 473 (w). MALDI-(+)MS [M]^+^ = 1167.462860 Da, calculated for C_60_H_76_N_2_P_2_O_8_Eu = 1167.42929 Da, Δ = 0.6 ppm C_60_H_76_N_2_P_2_O_8_Eu^+^ = (**9** − 2CH_3_OH − Cl^−^) (Figure S18).

### 3.4. Optical Measurements

Steady-state luminescence and excitation measurements in the visible and NIR regions were performed with a Fluorolog FL 3-22 spectrometer (Horiba-Jobin-Yvon, Edison, NJ, USA) which has a 450 W xenon lamp as the excitation source and an R-928 photomultiplier. All optical measurements were performed for powdered samples. Only quartz capillaries were used for luminescence measurements. Time-resolved experiments, namely, luminescence lanthanide lifetimes (τ), were measured with initial delay = 0.05 ms, step size = 0.02 ms, and final window = 10 ms were measured at least three times, which were achieved by monitoring the decay at the maxima of the emission spectra. For phosphorescence spectrum initial delay = 0.1 ms. The single- or bi-exponential decays were analyzed with Origin^®^ 8.1 (OriginLab Corporation, Northampton, MA, USA). The quantum yield measurements were carried out on solid samples with a Spectralone-covered G8 integration sphere (GMP SA, Renens, Switzerland) under ligand excitation, according to the absolute method of Wrighton [37,38,39]. Each sample was measured several times under slightly different experimental conditions. The estimated error for quantum yields was ±10%. The values of quantum yield are given in fraction.

### 3.5. Calculation Based on Luminescence Data

The intrinsic luminescence quantum yields and the radiative (k_r_) and nonradiative (k_nr_) rate constants were estimate using equations as follows, [16,40,41]

(1) $$\tau_{r}=\frac{1}{k_{r}}$$

(2) $$\tau_{\mathrm{obs}}=\frac{1}{k_{r}+k_{\mathrm{nr}}}$$

(3) $$Q_{\mathrm{Eu}}^{\mathrm{Eu}}=\frac{\tau_{\mathrm{obs}}}{\tau_{r}}=\tau_{\mathrm{obs}}\cdot A_{\mathrm{MD},0}\cdot n^{3}\cdot \left(\frac{I_{\mathrm{tot}}}{I_{\mathrm{MD},0}}\right)$$

where A_MD,0_ is the spontaneous luminescence probability for the ^5^D_0_ → ^7^F_1_ transition in vacuum (14.65 s^−1^), n is the refractive index of the medium, and (I_tot_/I_MD_) is the ratio of the total area of the Eu^3+^ luminescence spectrum to the area of the ^5^D_0_ → ^7^F_1_ transition band. n was taken as 1.5 for all cases [41].

The overall deactivation rate constant, which is inversely proportional to the lifetime τk_obs_ is given by

(4) $$k_{\mathrm{obs}}=k^{r}+\underset{n}{\sum }k_{n}^{\mathrm{nr}}=k^{r}+\underset{i}{\sum }k_{i}^{\mathrm{vibr}}\left(T\right)+\underset{j}{\sum }k_{j}^{\mathrm{pet}}\left(T\right)+\underset{k}{\sum }k_{k}^{\mathrm{Jnr}}$$

where k_r_ and k_nr_ are the radiative and non-radiative rate constants, respectively; the superscript “vibr” points to vibrational processes, while “pet” refers to photoinduced electron transfer processes; “jnr” are rate constants associated with remaining deactivation paths.

The energy of charge transfer state was estimated using by

*E*_CTS_ ≅ 30 000·[χ_opt_(X) − χ_uncorr_(Eu)] cm^−1^ (5)

where χ_opt_(X) and χ_uncorr_(Eu) are optical electronegativity of the ligand and uncorrected for spin correlation, respectively [16].

For energy gap calculations the energy of the ^5^D_0_ and ^5^D_4_ levels for Eu and Tb ions were taken as 17,300 and 20,500 cm^−1^ respectively.

## 4. Conclusions

The use of bis(diisopropylphenyl) phosphate-chloride ligand allowed to obtain a series of isomorphous heteroleptic complexes with a 7-coordinated Ln^3+^ cation. The interaction of complexes with terpyridine or phenanthroline leads to the formation of 1:1 adducts (Ln: N-containing ligand) with the simultaneous incorporation of water or methanol molecules into the coordination sphere of the Ln^3+^ ion. The luminescence intensity of organophosphosphate complexes themselves is low (quantum yields 0.01 and 0.1 for the Eu and Tb complexes); however, the introduction of N-containing ligand (terpyridine or phenanthroline) leads to a significant enhancement of luminescence ($Q_{Ln}^{Lig}$ 0.45 for Tb complex with terpyridine and 0.39 for Eu complex with phenanthroline). Moreover, the presence of two types of ligands in the coordination sphere (phosphate and phenanthroline) allows to design the Ln surrounding with very high intrinsic quantum yield $Q_{Eu}^{Eu}$ (0.92). It is worth emphasizing that a large volume of organophosphosphate ligand prevents the π-stacking interaction between N-containing ligands and relatively small coordination number (C.N. = 7) leads to shorter Ln-ligand bond lengths. These peculiarities of the organophosphosphate ligand promote high efficiency of the energy transfer process in the Ln complexes. In such a way, the performed study allows to conclude that diarylphosphate can be considered as a prospective ligand for the design of highly luminescent Ln complexes for both visible and NIR regions.

## Figures and Tables

**Table 1:** Selected bond lengths for **2**–**4** (Å).

	2 (Eu)	3 (Gd)	4 (Tb)
Ln-O1	2.2947 (10)	2.286 (2)	2.2669 (10)
Ln-O5	2.4366 (11)	2.422 (3)	2.4068 (12)
Ln-O6	2.4360 (12)	2.425 (3)	2.4086 (13)
Ln-Cl	2.6458 (6)	2.6443 (15)	2.6290 (6)

**Table 2:** Selected bond lengths for 5 (Å).

	Bond Length		Bond Length
Eu-O1	2.252 (5)	Eu-N1	2.558 (6)
Eu-O5	2.291 (5)	Eu-N3	2.565 (6)
Eu-O9	2.496 (6)	Eu-N2	2.601 (6)
Eu-O10	2.515 (6)	Eu-Cl	2.6850 (19)

**Table 3:** Selected bond lengths for **8** (Å).

	Bond Length		Bond Length
Nd-O1	2.3993 (16)	Nd-O14	2.5208 (16)
Nd-O5	2.3512 (16)	Nd-N1	2.6339 (19)
Nd-O9	2.3638 (16)	Nd-N2	2.6477 (19)
Nd-O13	2.5124 (17)	Nd-N3	2.599 (2)

**Table 4:** The ratio of the integrated intensities of the ^5^D_0_-^7^F*_J_* (*J* = 0, 2, 4) transitions of the Eu^3+^ ion to the integrated intensity of the ^5^D_0_ → ^7^F_1_ transition.

Complex	^5^D_0_-^7^F_0_	^5^D_0_-^7^F_2_	^5^D_0_-^7^F_4_	Total
**2**	0.05	6.55	1.87	9.89
**5**	0.03	3.26	1.03	5.51
**9**	0.01	6.64	0.97	8.87

**Table 5:** Energy of S1 and T1 states, observed lifetimes, values of energy gaps E1 and E2, as well as intrinsic ($Q_{Eu}^{Eu}$) and overall ($Q_{Eu}^{Lig})$ quantum yields ^a^.

	τ_obs_, ms	S_1_, nm/cm^−1^	T, nm/cm^−1^	E_1_, cm^−1^	E_2_, cm^−1^	$Q_{Eu}^{Eu}{\;}^{b}$	$Q_{Eu}^{Lig}$
**2**	0.28 ± 0.01	275/36,350	395/25,300	11050	8100	0.13	0.01
**5**	0.61 ± 0.02	355/28,170	440/22,730	5440	5530	0.17	0.11
**9**	1.77 ± 0.02	350/28,570	450/22,200	6370	5000	0.92	0.39

^a^ All photophysical data were measured at ambient temperature; ^b^ intrinsic quantum yield was calculated by Equation (3), values of quantum yield are given in fraction.

## Supplementary Materials

The Supplementary Materials are available online. Figure S1. Luminescence excitation spectrum of Eu complex **5** at 77 K, λ_reg_ = 615 nm; Figure S2. Luminescence spectra of **Tb** complex (**4**) at 300 (a) and 77 K (b), λ_exc_ = 320 nm; Figure S3. Luminescence spectra of **Tb** complex (**7**) at 300 (a) and 77 K (b), λ_exc_ = 320 nm; Figure S4. Luminescence spectra of **Nd** complex (**8**) at 300 (a) and 77 K (b), λ_exc_ = 320 nm; Figure S5. Phosphorescence spectrum of **Gd** complex (**6**) at 77 K, λ_exc_ = 320 nm. *—transitions due to residual traces of Eu and Tb; Figure S6. Crystal structure of (**2**), (**3**) and (**4**), and hydrogen bonding; Figure S7. Crystal structure of (**5**); Figure S8. Hydrogen bonding in (**5**); Figure S9. Crystal structure of (**8**) and hydrogen bonding; Figure S10. IR spectrum of **5**; Figure S11. IR spectrum of **6**; Figure S12. IR spectrum of **7**; Figure S13. IR spectrum of **8**; Figure S13a. IR spectrum of **8** obtained from [(O_2_P(OAr)_2_)_3_Nd(MeOH)_5_] (route B). (measured on a Ostec Ft-IR spectrometer); Figure S14. IR spectrum of **9**; Figure S15. Experimentally detected and theoretical MALDI-(+)MS spectrum of **5**; main experimental peak [M]^+^ = 1220.46074 Da, calculated for C_63_H_79_N_3_P_2_O_8_Eu = 1220.45492 Da, Δ = 4.8 ppm. C_63_H_79_N_3_P_2_O_8_Eu = (**5** − 2H_2_O-CH_3_OH − Cl^−^); Figure S16. Experimentally detected and theoretical MALDI-(+)MS spectrum of (**6**); main experimental peak [M]^+^ = 1224.45889 Da, calculated for C_63_H_79_N_3_P_2_O_8_Gd = 1224.45839 Da, Δ = 0.4 ppm. C_63_H_79_N_3_P_2_O_8_Gd = (**6** − 2H_2_O-CH_3_OH − Cl^−^); Figure S17. Experimentally detected and theoretical MALDI-(+)MS spectrum of (**7**); main experimental peak [M]^+^ = 1226.46134 Da, calculated for C_63_H_79_N_3_P_2_O_8_Tb = 1226.45905 Da, Δ = 1.8 ppm. C_63_H_79_N_3_P_2_O_8_Tb = (**7** − 2H_2_O-CH_3_OH − Cl^−^); Figure S18. Experimentally detected and theoretical MALDI-(+)MS spectrum of (**9**); main experimental peak [M]^+^ = 1167.462860 Da, calculated for C_60_H_76_N_2_P_2_O_8_Eu = 1167.42929 Da, Δ = 0.6 ppm. C_60_H_76_N_2_P_2_O_8_Eu+ = (**9** − 2CH_3_OH − Cl^−^); Table S1. X-ray crystallographic data and refinement details for studied complexes; Table S2. Selected bond distances (Å) in (**2**), (**3**) and (**4**); Table S3. Hydrogen-bond geometry (Å, °) for (**2**), (**3**) and (**4**); Table S4. Selected bond distances (Å) in (**5**); Table S5. Hydrogen-bond geometry (Å, °) for (**5**); Table S6. Selected bond distances (Å) in (**8**); Table S7. Hydrogen-bond geometry (Å, °) for (**8**).
