# Supplementary material for: Diarylphosphate as a New Route for Design of Highly Luminescent Ln Complexes
Source: Molecules. 2020 Aug 28;25(17):3934. doi: 10.3390/molecules25173934 (PMC7504456; doi:10.3390/molecules25173934)
Supplement: Supplementary file 1 [file molecules-25-03934-s001.pdf]

Electronic Supporting information

Diarylphosphate as a New Route for Design of Highly Luminescent Ln Complexes

Alexey E. Kalugin [1,2], Mikhail E. Minyaev [1,3], Lada N. Puntus [1,4], Ilya V. Taydakov [5], Evgenia A. Varaksina [1,5], Konstantin A. Lyssenko [6], Ilya E. Nifant'ev [1,6] and Dmitrii M. Roitershtein [1,3,7,*]

[1] A.V. Topchiev Institute of Petrochemical Synthesis RAS, 119991 Moscow, Russia; alex.kalug@gmail.com (A.E.K.); mminyaev@mail.ru (M.E.M.); ladapuntus@gmail.com (L.N.P.); janiy92@yandex.ru (E.A.V.); inif@org.chem.msu.ru (I.E.N.)
[2] Moscow Institute of Physics and Technology (MIPT), 141701 Dolgoprudnyi, Moscow Region, Russia
[3] N.D. Zelinsky Institute of Organic Chemistry, RAS, 119991 Moscow, Russia
[4] V.A. Kotel'nikov Institute of Radioengineering and Electronics, RAS, 141190 Fryazino, Moscow Region, Russia
[5] P.N. Lebedev Physical Institute, RAS, 119991 Moscow, Russia; taidakov@gmail.com
[6] Chemistry Department, M.V. Lomonosov Moscow State University, 119991 Moscow, Russia; kostya@xray.ineos.ac.ru
[7] National Research University Higher School of Economics, 101000 Moscow, Russia
* Correspondence: roiter@yandex.ru; Tel.: +7-916-373-3507

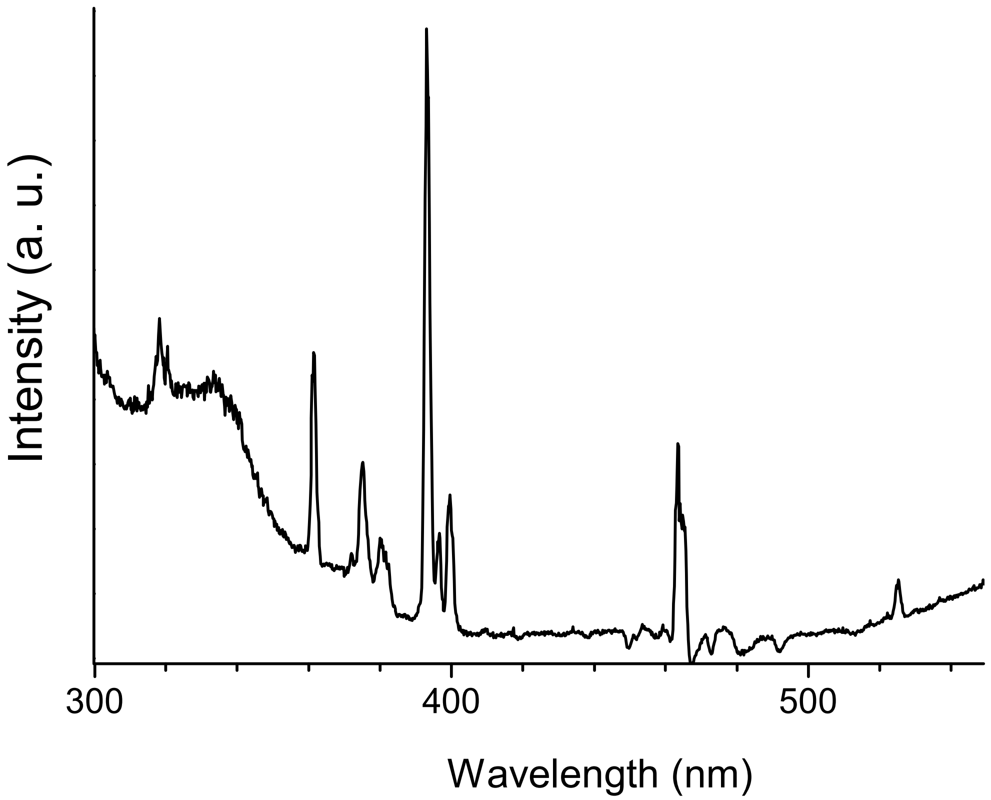

Figure S1. Luminescence excitation spectrum of Eu complex **5** at 77 K, λ_{reg} = 615 nm

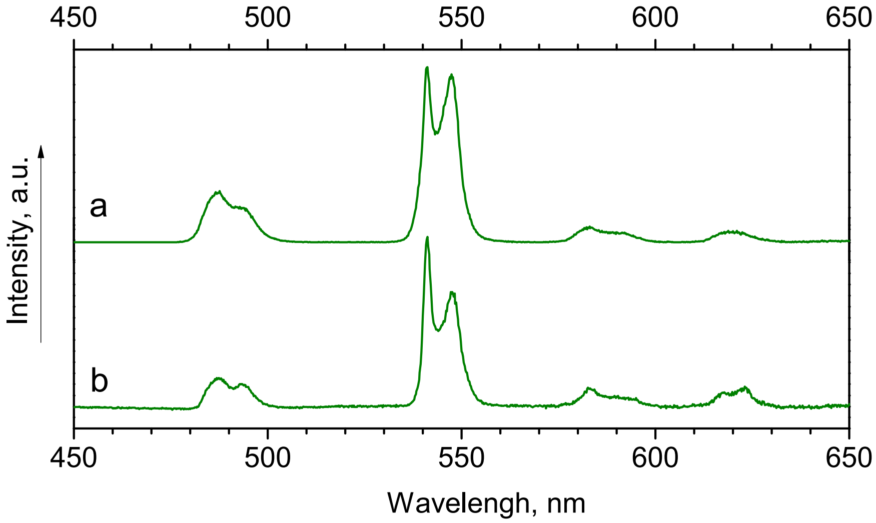

Figure S2. Luminescence spectra of **Tb** complex (**4**) at 300 (a) and 77 K (b), λ_{exc} = 320 nm.

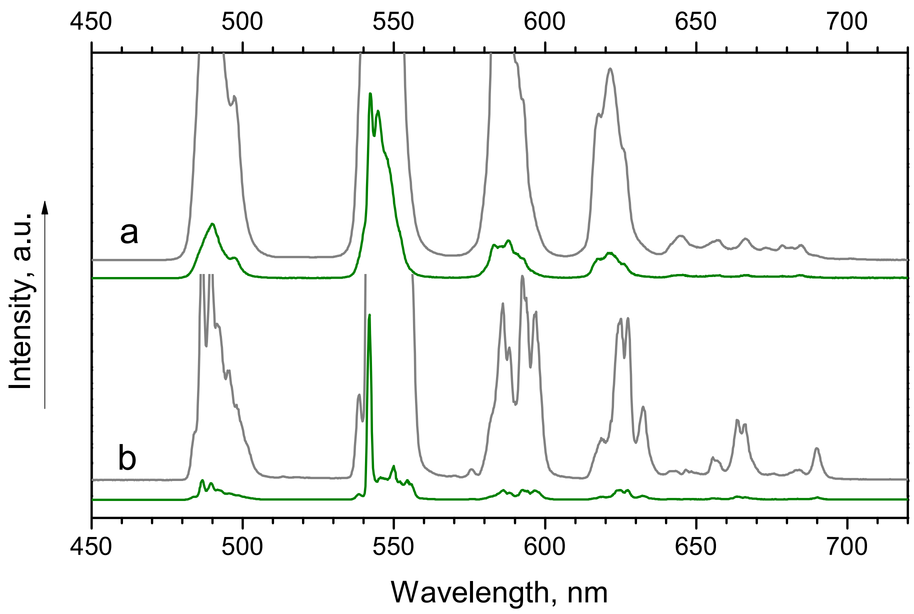

Figure S3. Luminescence spectra of **Tb** complex (**7**) at 300 (a) and 77 K (b), λ_{exc} = 320 nm.

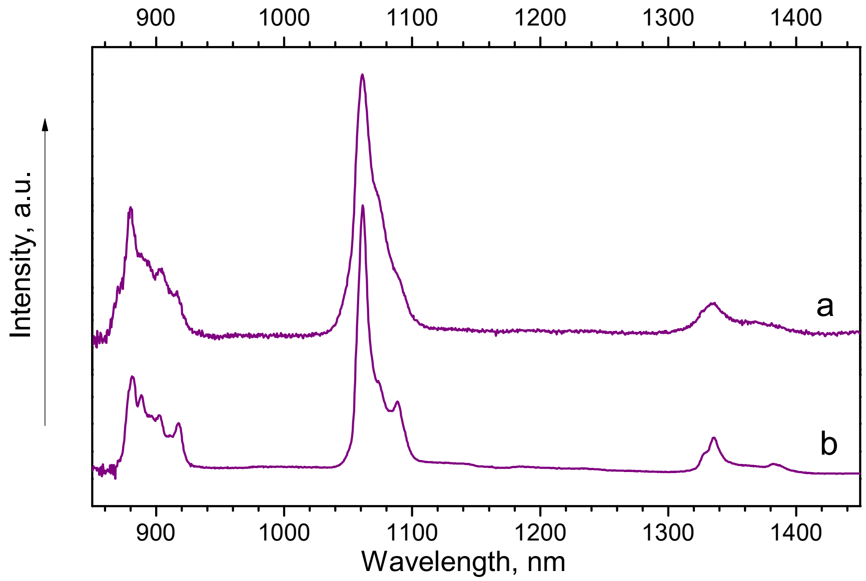

Figure S4. Luminescence spectra of **Nd** complex (**8**) at 300 (a) and 77 K (b), λ_{exc} = 320 nm

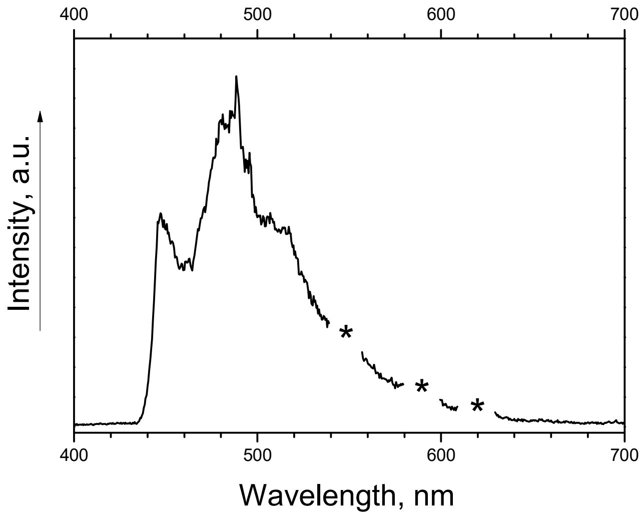

Figure S5. Phosphorescence spectrum of **Gd** complex (**6**) at 77 K, λ_{exc} = 320 nm. * - transitions due to residual traces of Eu and Tb.

X-ray diffraction data

Table S1. X-ray crystallographic data and refinement details for studied complexes.

	**2**	**3**	**4**	**5**	**8**
Empirical formula	$C_{52}H_{84}ClEuO_{12}P_2 \cdot 2(CH_4O)$	$C_{52}H_{84}ClGdO_{12}P_2 \cdot 2(CH_4O)$	$C_{52}H_{84}ClO_{12}P_2Tb \cdot 2(CH_4O)$	$C_{63}H_{83}ClEuN_3O_{10}P_2 \cdot CH_4O$	$C_{88}H_{119}N_3NdO_{14}P_3 \cdot C_7H_8 \cdot C_6H_{14}$
Formula weight	1214.62	1219.91	1221.58	1323.71	1858.31
Crystal system	Monoclinic	Monoclinic	Monoclinic	Triclinic	Orthorhombic
Space group	*C*2/*c*	*C*2/*c*	*C*2/*c*	$P\bar{1}$	*Pbca*
Temperature (K)	120	100	100	120	120
Unit cell dimensions					
a (Å)	16.9488 (7)	16.9393 (11)	16.9334 (5)	11.292(2)	27.6119(7)
b (Å)	12.3857 (5)	12.4094 (8)	12.3945 (4)	16.740(4)	20.8417(6)
c (Å)	30.4515 (12)	30.458 (2)	30.4167 (10)	18.225(3)	34.1800(9)
α (°)	90	90	90	91.670(4)	90
β (°)	104.8579 (8)	104.7549 (19)	104.764 (1)	90.424(3)	90
γ (°)	90	90	90	109.426(3)	90
Volume ($Å^3$)	6178.7 (4)	6191.3 (7)	6173.1 (3)	3247.0(11)	19669.9(9)
Z	4	4	4	2	8
d_{calcd}(g/cm^3)	1.306	1.309	1.314	1.354	1.255
μ (mm^{-1})	1.17	1.22	1.30	1.12	0.64
F(000)	2552	2556	2560	1380	7880
θ Range (°)	2.06-30.04	2.06-27.00	1.38-33.65	1.12-28.00	1.48-28.89
Completeness to θ_{max}	1.000	0.996	0.956	0.988	0.999
Refl. collected	65697	14308	76214	34110	231465
Refl. unique (R_{int})	9052 (0.0381)	6738 (0.0598)	11719 (0.0501)	15486 (0.0699)	25845 (0.0811)
Refl. with $I > 2\sigma(I)$	8519	5358	10553	12097	18981
Variables / restraints	349 / 1	349 / 0	349 / 0	757 / 0	1050 / 6
Goodness-of-fit on F^2	1.083	1.035	1.054	1.057	1.007
Final R1, wR2 indices with $I>2\sigma(I)$	0.0236, 0.0552	0.0477, 0.0866_	0.0297, 0.0612	0.0678, 0.0926	0.0381, 0.0827
Final R1, wR2 indices (all data)	0.0261, 0.0563	0.0656, 0.0937	0.0367, 0.0638	0.1645, 0.1855	0.0627, 0.0949
$\Delta\rho_{max}$, $\Delta\rho_{min}$ (e $Å^{-3}$)	0.54, −0.55	0.95, −1.50	0.51, −0.54	1.83, -2.42	1.14, -0.72

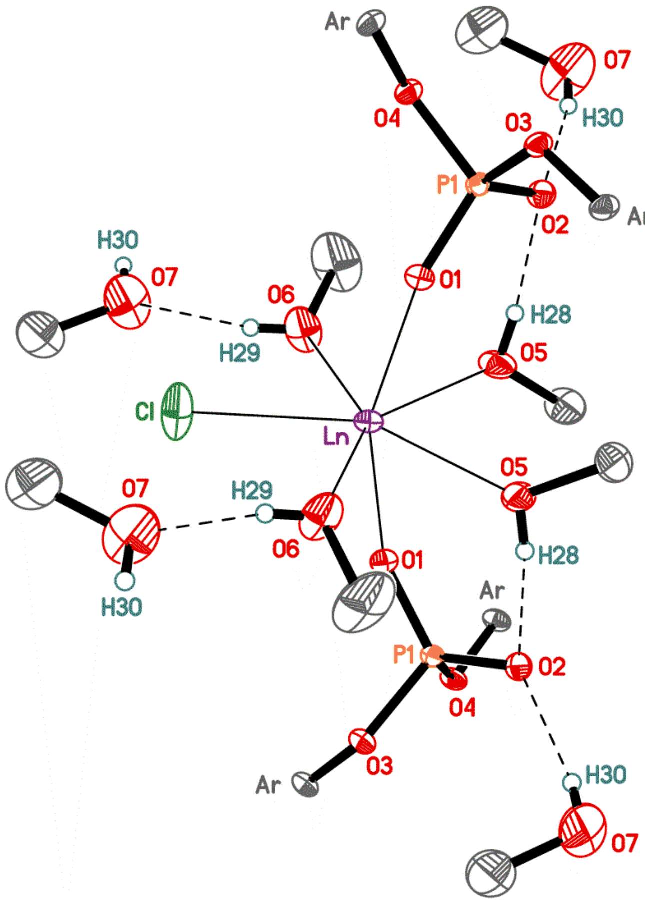

Figure S6. Crystal structure of (**2**), (**3**) and (**4**), and hydrogen bonding.

Table S2. Selected bond distances (Å) in (**2**), (**3**) and (**4**).

Bond	(2), Eu	(3), Gd	(4), Tb	Bond	(2), Eu	(3), Gd	(4), Tb
Ln-Cl	2.6458(6)	2.6443(15)	2.6290(6)	P1-O1	1.5075(10)	1.506(3)	1.5068(10)
Ln-O1	2.2947(10)	2.286(2)	2.2669(10)	P1-O2	1.4925(10)	1.490(3)	1.4914(10)
Ln-O5	2.4366(11)	2.422(3)	2.4068(12)	P1-O3	1.5870(10)	1.586(2)	1.5860(10)
Ln-O6	2.4360(12)	2.425(3)	2.4086(13)	P1-O4	1.5882(10)	1.587(3)	1.5877(10)

Table S3. Hydrogen-bond geometry (Å, º) for (**2**), (**3**) and (**4**).

Complex	*D*—H···*A*	*D*—H	H···*A*	*D*···*A*	*D*—H···*A*
(**2**), Ln=Eu	O5—H28···O2	0.79 (2)	1.84 (2)	2.6242 (15)	169 (2)
	O7—H30···O2	0.78 (3)	2.03 (3)	2.8000 (18)	169 (3)
	O6—H29···O7[i]	0.76 (2)	2.05 (2)	2.777 (2)	160 (3)
	O6—H29···Cl1	0.76 (2)	2.67 (3)	2.9575 (13)	105 (2)
(**3**), Ln=Gd	O5—H28···O2	0.82 (4)	1.81 (4)	2.628 (4)	174 (4)
	O7—H30···O2	0.80 (7)	2.01 (7)	2.803 (5)	168 (7)
	O6—H29···O7[i]	0.81 (5)	2.03 (5)	2.788 (5)	157 (5)
	O6—H29···Cl1	0.81 (5)	2.63 (5)	2.954 (3)	106 (5)
(**4**), Ln=Tb	O5—H28···O2	0.83 (2)	1.80 (2)	2.6185 (16)	167 (2)
	O7—H30···O2	0.79 (3)	2.03 (3)	2.8046 (19)	166 (3)
	O6—H29···O7[i]	0.75 (3)	2.09 (3)	2.801 (2)	157 (3)
	O6—H29···Cl1	0.75 (3)	2.63 (3)	2.9370 (14)	107 (2)

Symmetry code: (i) $-x+3/2$, $y-1/2$, $-z+1/2$.

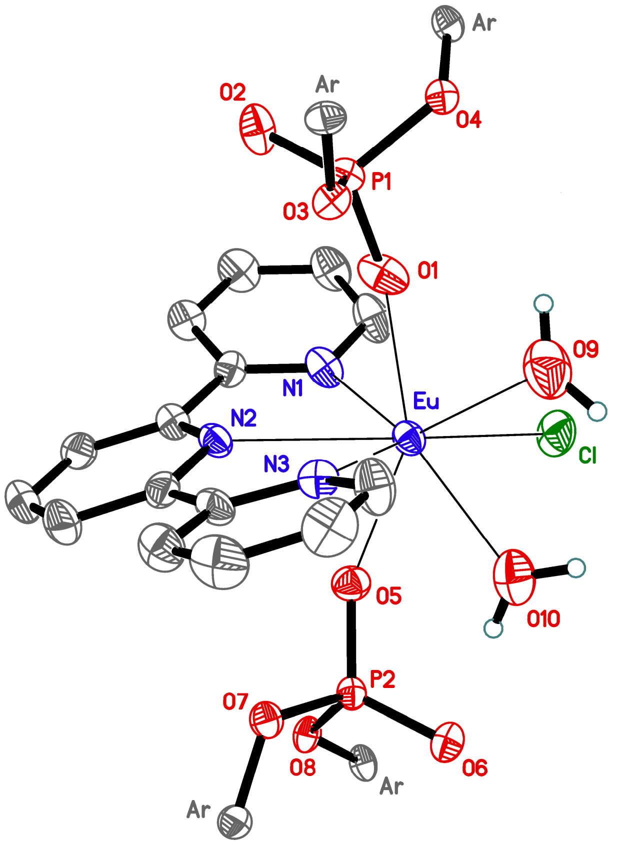

Figure S7. Crystal structure of (**5**).

Table S4. Selected bond distances (Å) in (**5**).

Eu—O1	2.252 (5)	Eu—N1	2.558 (6)	P1—O1	1.499 (5)	P2—O5	1.502 (5)
Eu—O5	2.291 (5)	Eu—N3	2.565 (6)	P1—O2	1.464 (6)	P2—O6	1.483 (5)
Eu—O9	2.496 (6)	Eu—N2	2.601 (6)	P1—O3	1.614 (5)	P2—O7	1.594 (5)
Eu—O10	2.515 (6)	Eu—Cl1	2.6850 (19)	P1—O4	1.608 (5)	P2—O8	1.593)

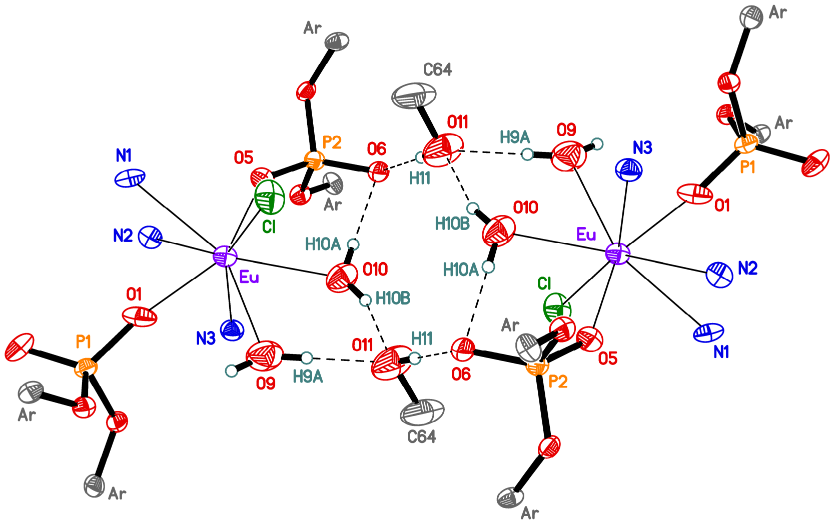

Figure S8. Hydrogen bonding in (**5**).

Table S5. Hydrogen-bond geometry (Å, º) for (**5**).

D—H···*A*	*D*—H	H···*A*	*D*···*A*	*D*—H···*A*
O9—H9A···O11	0.85	1.75	2.597 (11)	179.8
O10—H10A···O6	0.85	1.94	2.792 (8)	179.8
O10—H10B···O11	0.85	1.91	2.725 (9)	161.3
O11—H11···O6^{i}	0.84	1.79	2.637 (8)	179.1

Symmetry code: (i) −*x*+1, −*y*+1, −*z*+2.

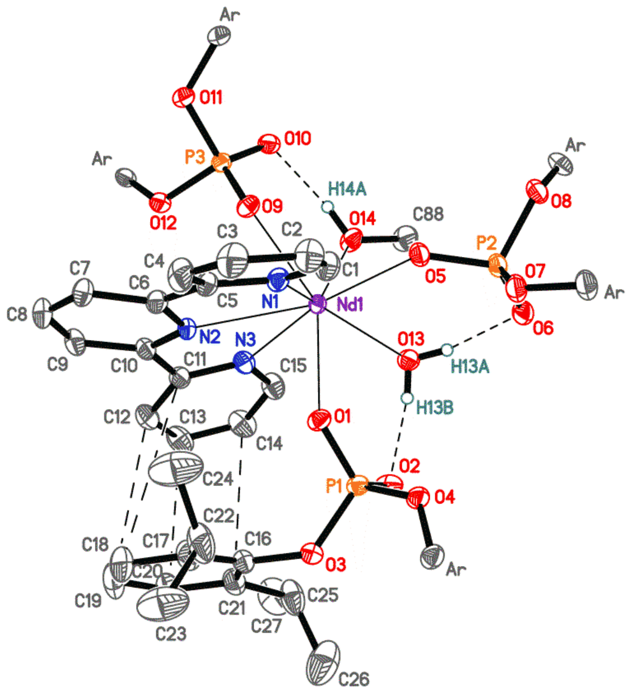

Figure S9. Crystal structure of (**8**) and hydrogen bonding.

Table S6. Selected bond distances (Å) in (**8**).

Nd-O1	2.3993(16)	Nd-N1	2.6339(19)	P1-O3	1.5991(17)	P2-O8	1.6052(17)
Nd-O5	2.3512(16)	Nd-N2	2.6477(19)	P1-O4	1.5920(18)	P3-O9	1.4982(17)
Nd-O9	2.3638(16)	Nd-N3	2.599(2)	P2-O5	1.5053(17)	P3-O10	1.4834(17)
Nd-O13	2.5124(17)	P1-O1	1.5022(17)	P2-O6	1.4800(18)	P3-O11	1.5981(17)
Nd-O14	2.5208(16)	P1-O2	1.4878(17)	P2-O7	1.5971(18)	P3-O12	1.5981(17)

Table S7. Hydrogen-bond geometry (Å, º) for (**8**).

D—H···*A*	*D*—H	H···*A*	*D*···*A*	*D*—H···*A*
O13—H13*B*···O2	0.77 (3)	1.83 (3)	2.589 (3)	169 (3)
O13—H13*A*···O6	0.83 (3)	1.88 (3)	2.692 (3)	164 (3)
O14—H14*A*···O10	0.86	1.77	2.620 (2)	168

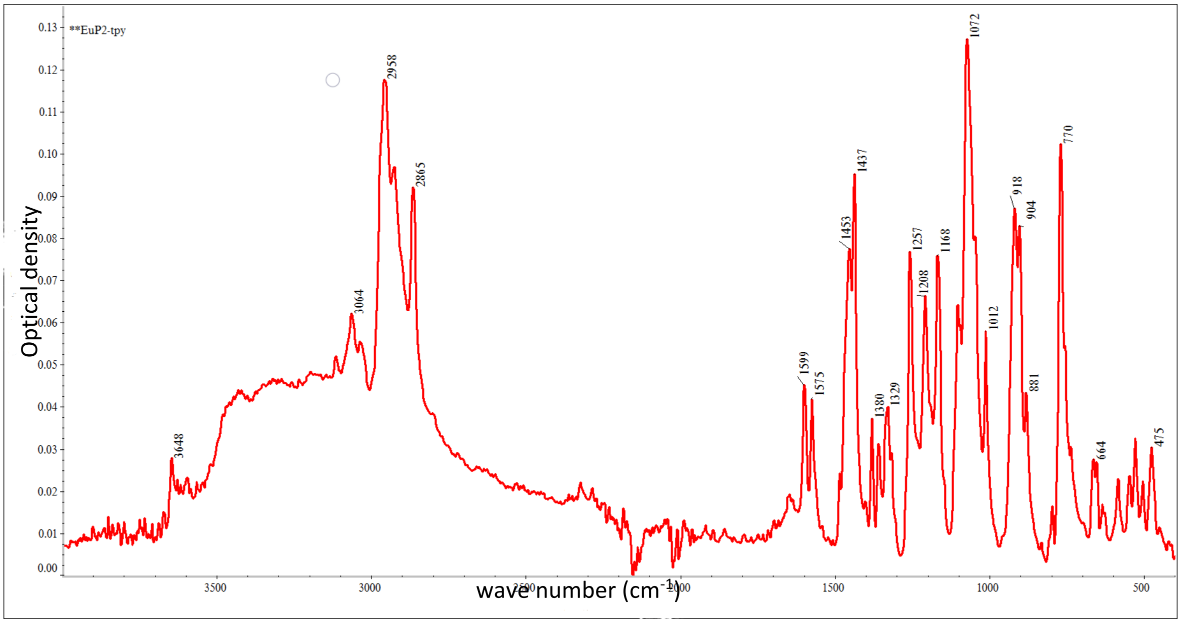

Figure S10. IR spectrum of **5**.

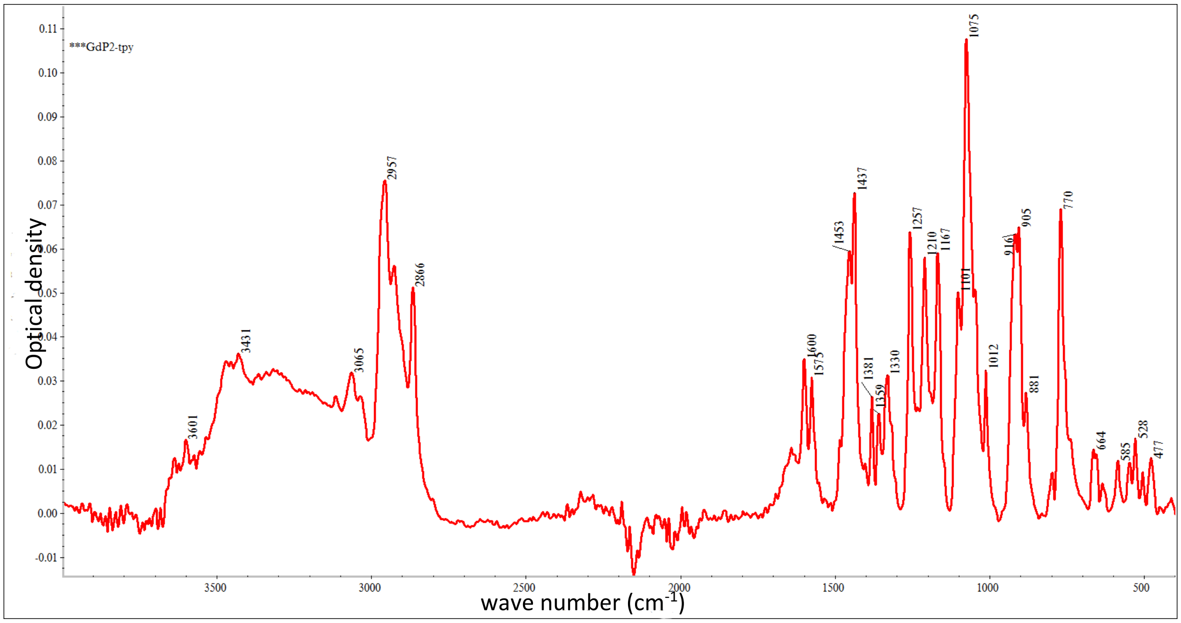

Figure S11. IR spectrum of **6**.

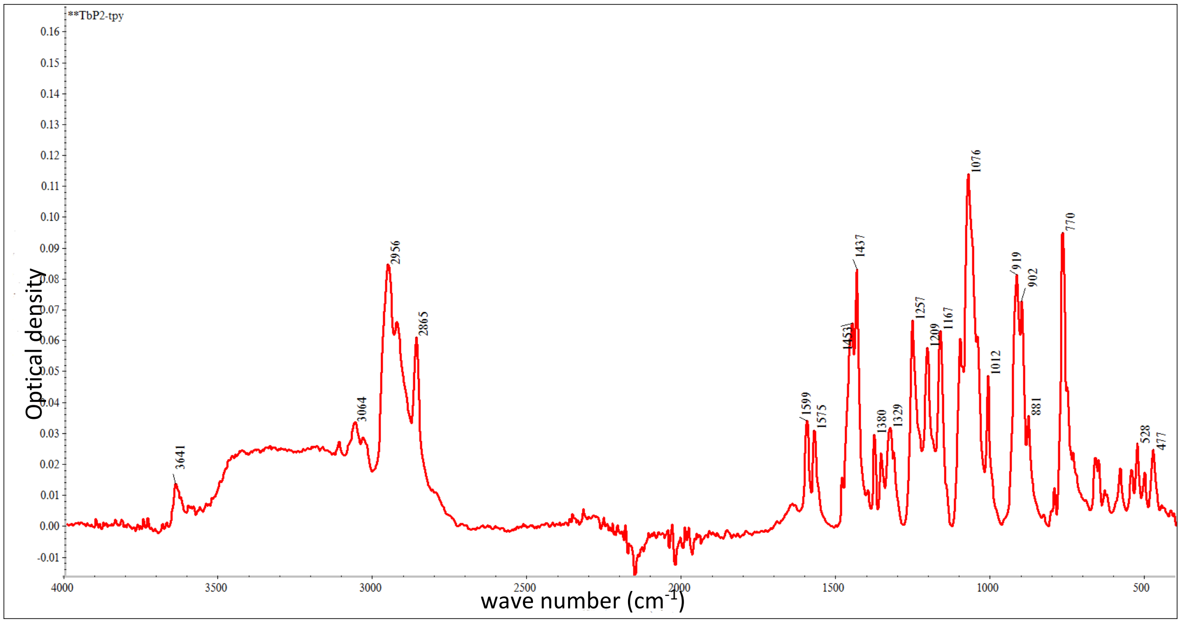

Figure S12. IR spectrum of **7**.

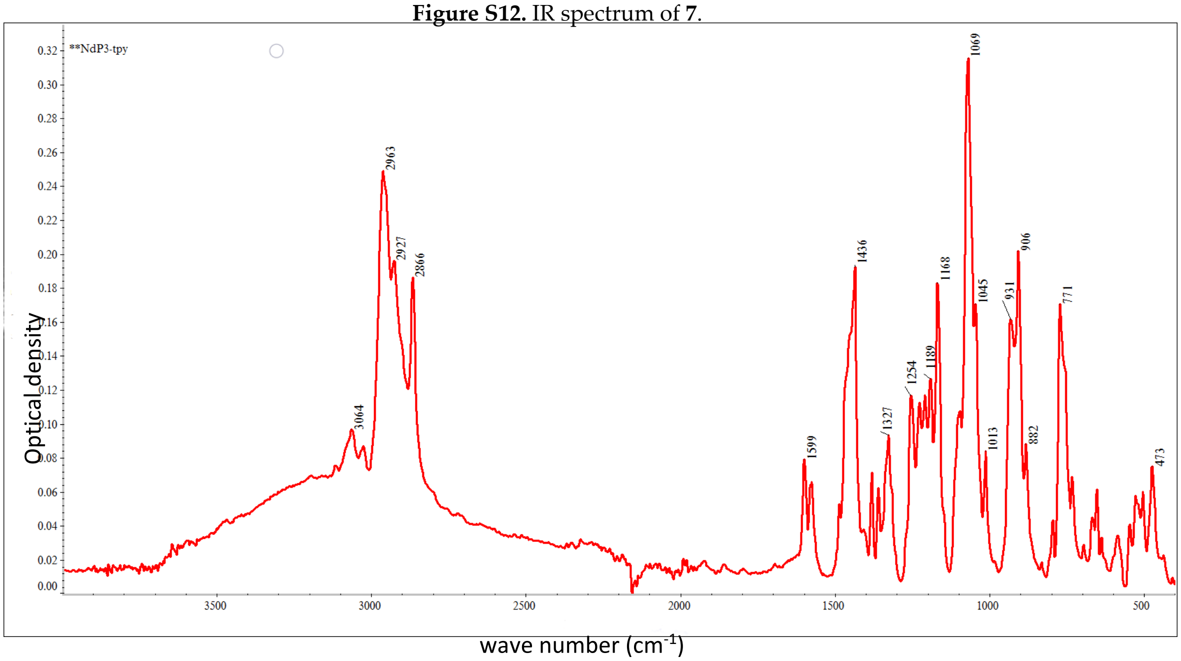

Figure S13. IR spectrum of **8**.

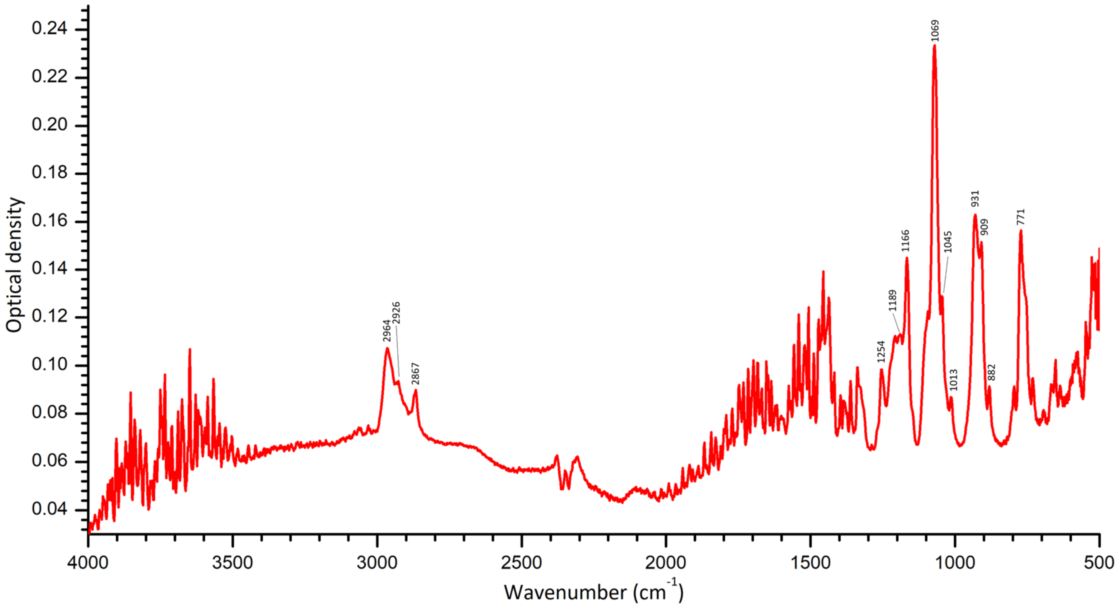

Figure S13a. IR spectrum of **8** obtained from $[(O_2P(OAr)_2)_3Nd(MeOH)_5]$ (route B). (measured on a Ostec Ft-IR spectrometer).

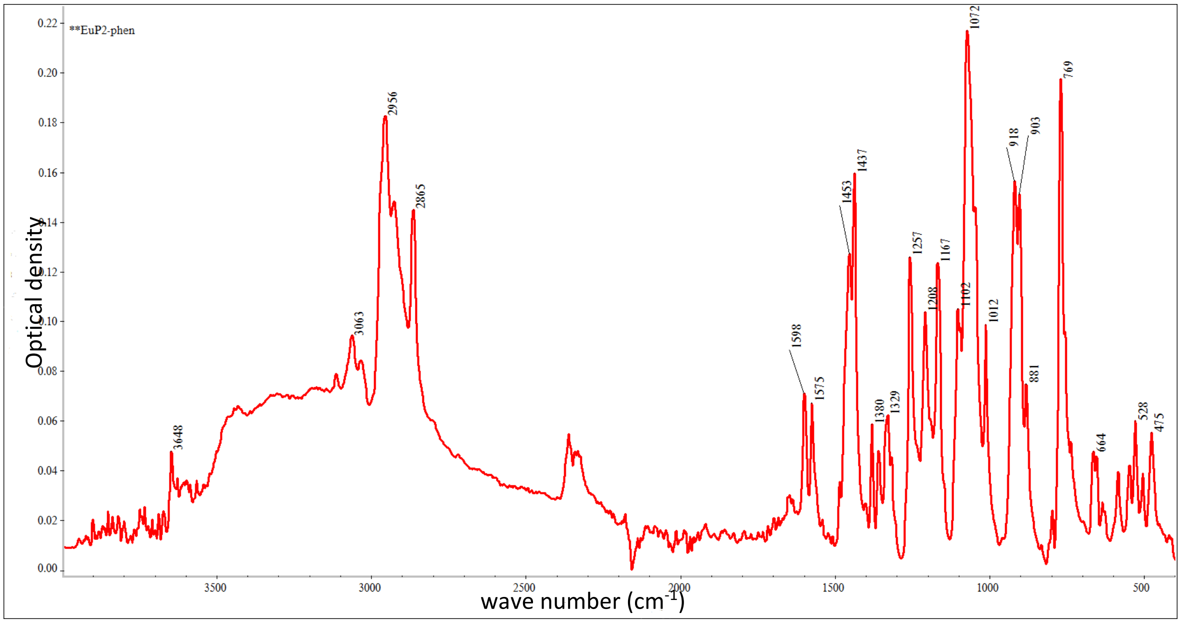

Figure S14. IR spectrum of **9**.

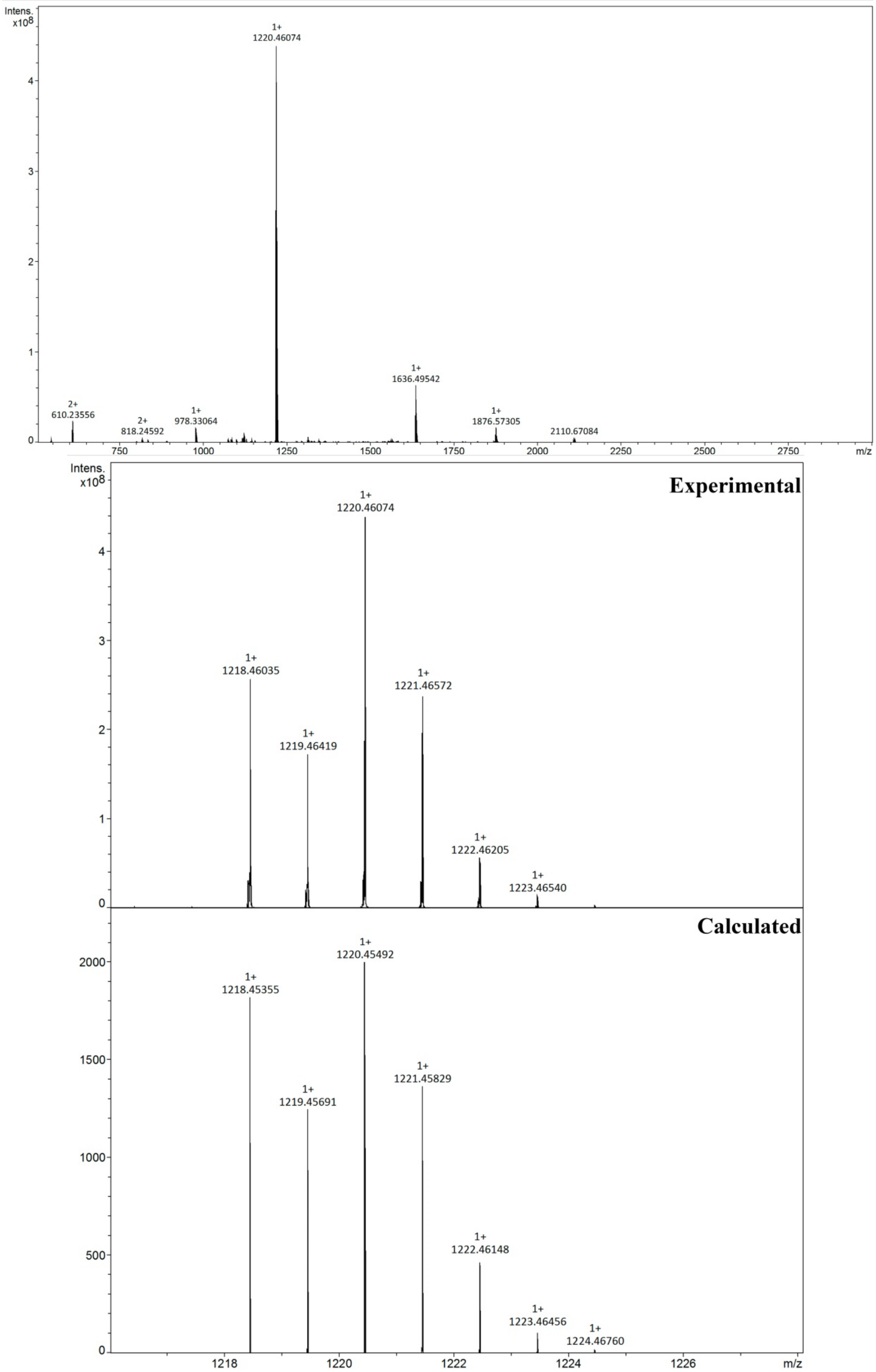

Figure S15. Experimentally detected and theoretical MALDI-(+)MS spectrum of **5**; main experimental peak $[M]^+$= 1220.46074 Da, calculated for $C_{63}H_{79}N_3P_2O_8Eu$ = 1220.45492 Da, Δ = 4.8 ppm. $C_{63}H_{79}N_3P_2O_8Eu$ = (**5** – $2H_2O$-CH_3OH – Cl^-).

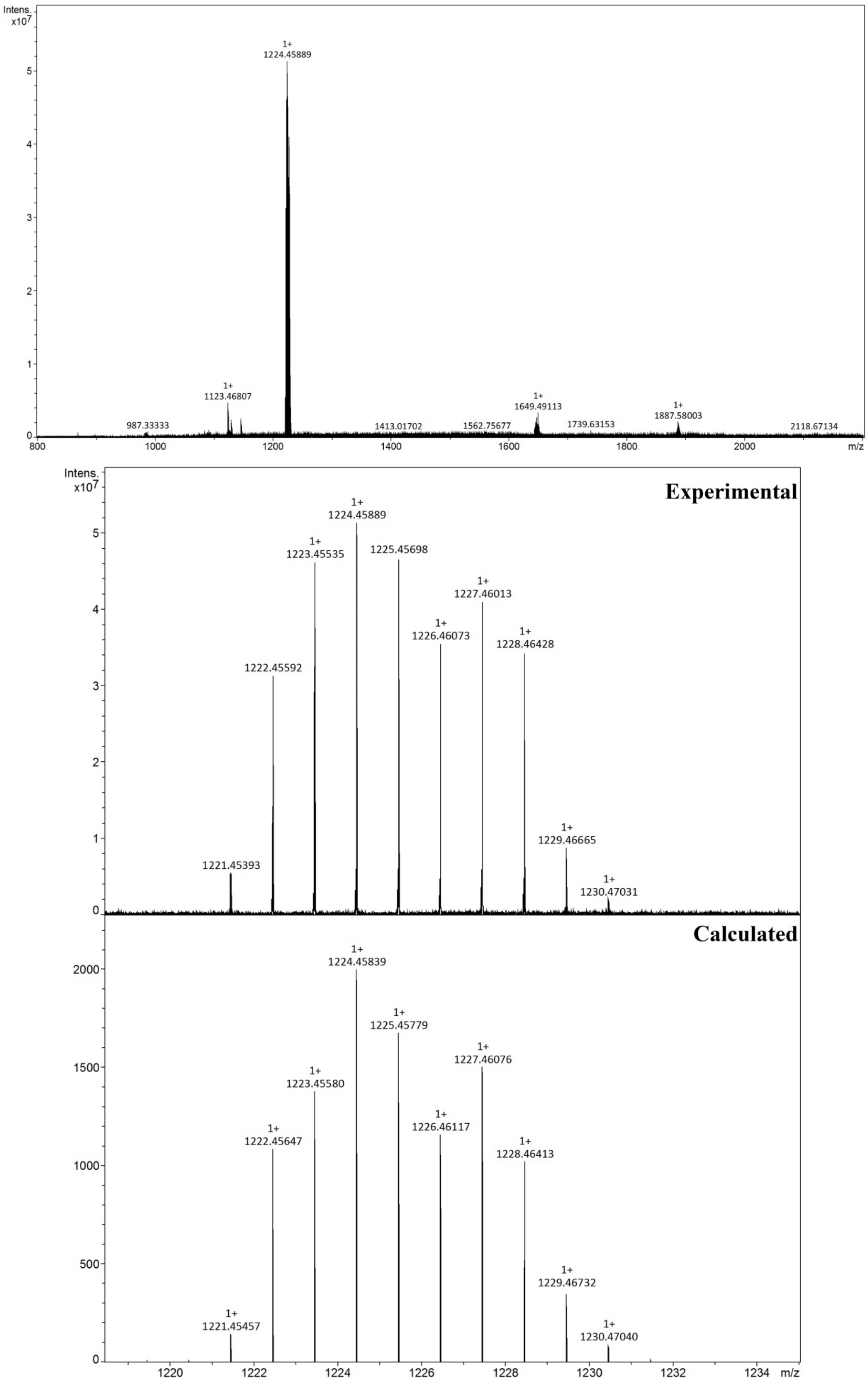

Figure S16. Experimentally detected and theoretical MALDI-(+)MS spectrum of **(6)**; main experimental peak $[M]^+$= 1224.45889 Da, calculated for $C_{63}H_{79}N_3P_2O_8Gd$ = 1224.45839 Da, Δ = 0.4 ppm. $C_{63}H_{79}N_3P_2O_8Gd$ = (**6** – $2H_2O$-CH_3OH – Cl^-).

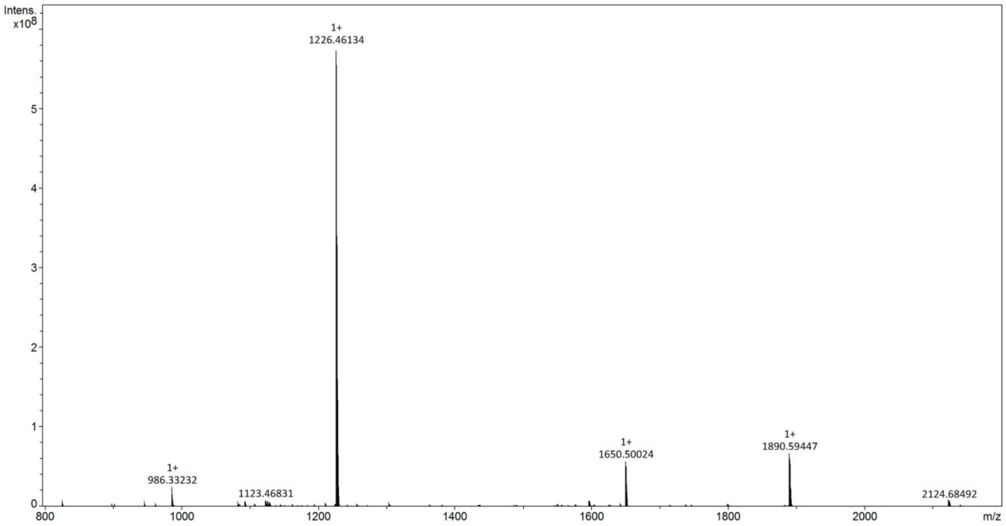
Intens.
x10^8
1+
1226.46134
1+
986.33232
1123.46831
1+
1650.50024
1+
1890.59447
2124.68492
m/z

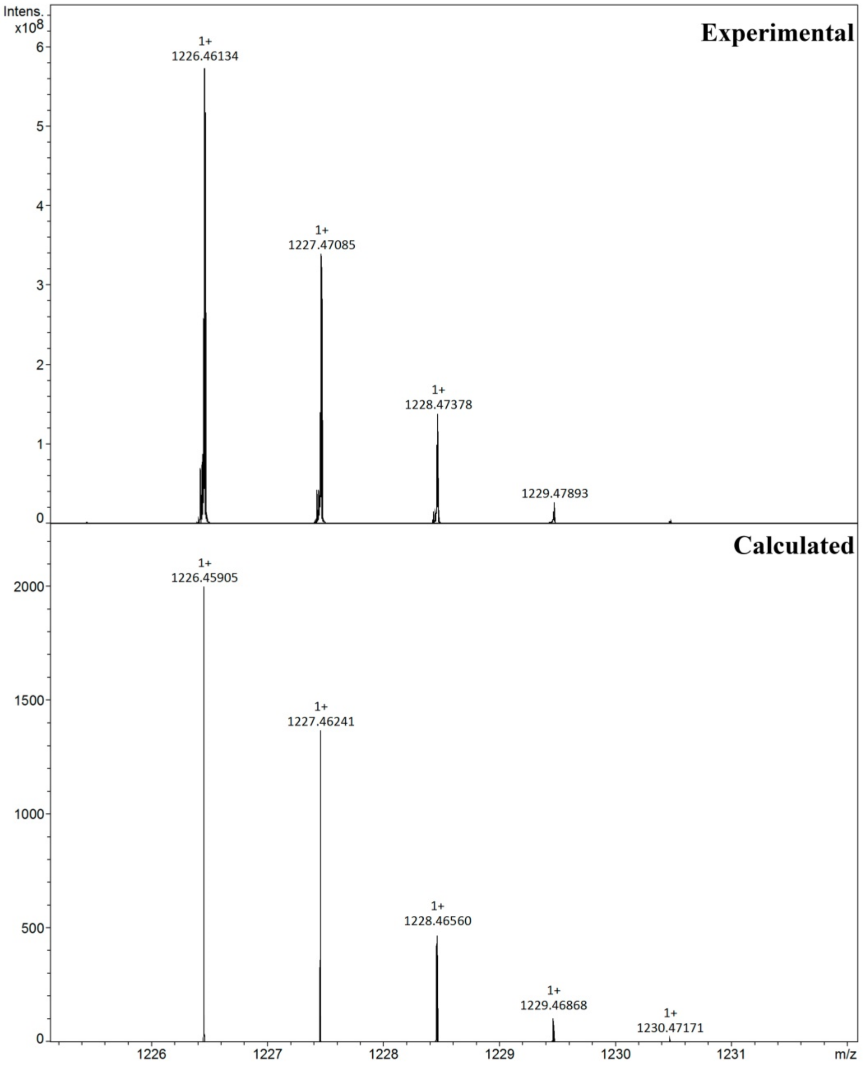
Intens.
x10^8
Experimental
1+
1226.46134
1+
1227.47085
1+
1228.47378
1229.47893
Calculated
1+
1226.45905
1+
1227.46241
1+
1228.46560
1+
1229.46868
1+
1230.47171
m/z

Figure S17. Experimentally detected and theoretical MALDI-(+)MS spectrum of **(7)**; main experimental peak $[M]^+$= 1226.46134 Da, calculated for $C_{63}H_{79}N_3P_2O_8Tb$ = 1226.45905 Da, Δ = 1.8 ppm. $C_{63}H_{79}N_3P_2O_8Tb$ = (**7** – $2H_2O$-CH_3OH – Cl^-).

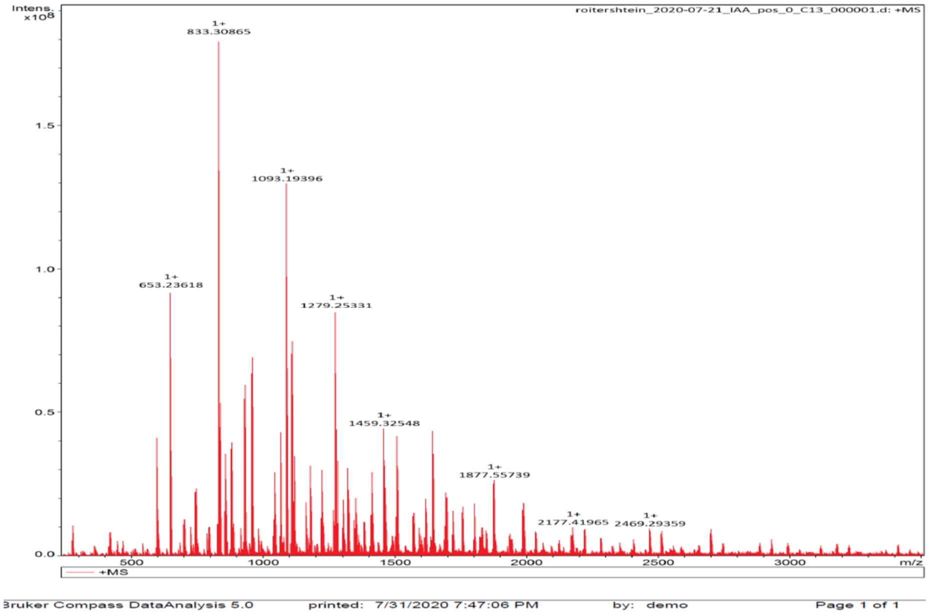

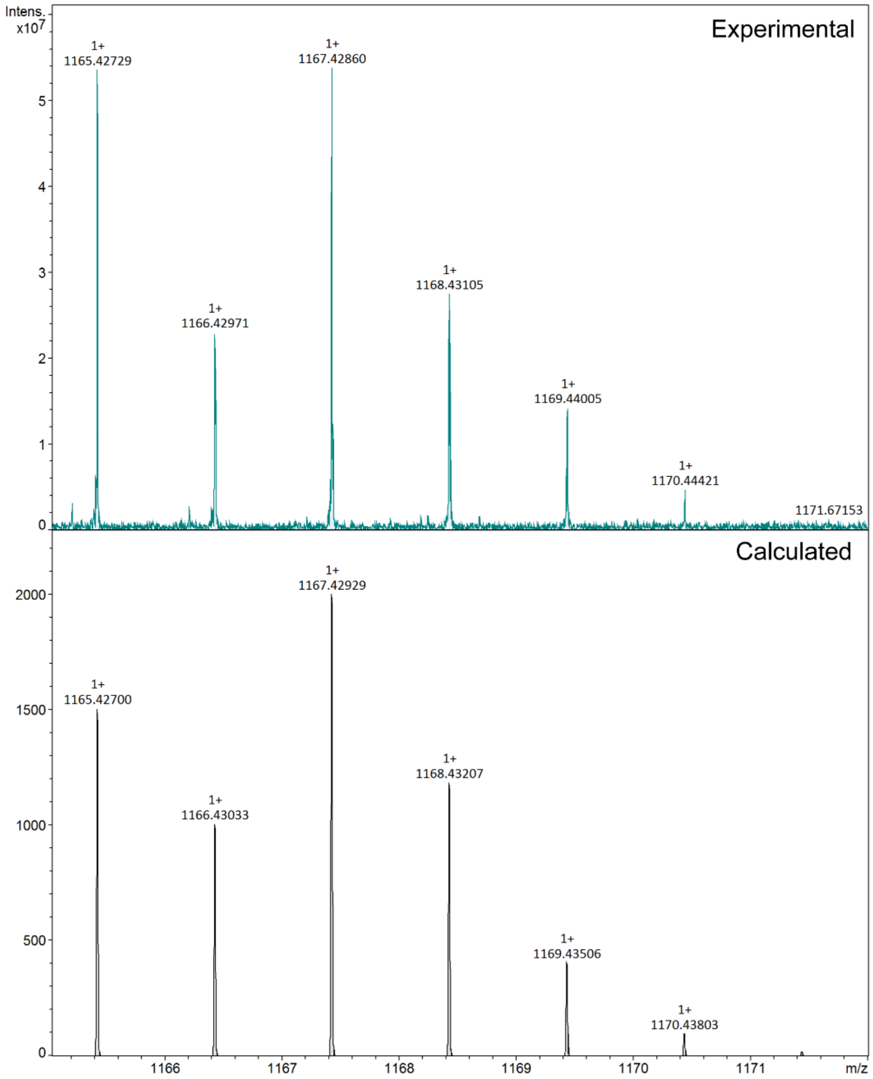

Figure S18. Experimentally detected and theoretical MALDI-(+)MS spectrum of **(9)**; main experimental peak $[M]^+$= 1167.462860 Da, calculated for $C_{60}H_{76}N_2P_2O_8Eu$ = 1167.42929 Da, Δ = 0.6 ppm. $C_{60}H_{76}N_2P_2O_8Eu^+$ = (**9** – $2CH_3OH$ – Cl^-).
